# Cytoplasmic Citrate Flux Modulates the Immune Stimulatory NKG2D Ligand MICA in Cancer Cells

**DOI:** 10.3389/fimmu.2020.01968

**Published:** 2020-08-11

**Authors:** Sofie H. Møller, Maiken Mellergaard, Mikkel Madsen, Amaia V. Bermejo, Stine D. Jepsen, Marie H. Hansen, Rikke I. Høgh, Blanca I. Aldana, Claus Desler, Lene Juel Rasmussen, Elahu G. Sustarsic, Zachary Gerhart-Hines, Evangelia Daskalaki, Craig E. Wheelock, Thomas K. Hiron, Da Lin, Christopher A. O’Callaghan, Hans H. Wandall, Lars Andresen, Søren Skov

**Affiliations:** ^1^Department of Veterinary and Animal Sciences, University of Copenhagen, Frederiksberg, Denmark; ^2^Department of Drug Design and Pharmacology, University of Copenhagen, Copenhagen, Denmark; ^3^Center for Healthy Aging, Department of Cellular and Molecular Medicine, University of Copenhagen, Copenhagen, Denmark; ^4^Novo Nordisk Foundation Center for Basic Metabolic Research, University of Copenhagen, Copenhagen, Denmark; ^5^Department of Medical Biochemistry and Biophysics, Karolinska Institutet, Stockholm, Sweden; ^6^Wellcome Trust Centre for Human Genetics, University of Oxford, Oxford, United Kingdom; ^7^Copenhagen Center for Glycomics, Department of Cellular and Molecular Medicine, University of Copenhagen, Copenhagen, Denmark

**Keywords:** cancer metabolism, tumor immunology, MHC class I chain-related proteins A, citrate, ATP citrate lyase, Natural Killer Group 2D

## Abstract

Immune surveillance of cancer cells is facilitated by the Natural Killer Group 2D (NKG2D) receptor expressed by different lymphocyte subsets. It recognizes NKG2D ligands that are rarely expressed on healthy cells, but upregulated by tumorigenesis, presenting a target for immunological clearance. The molecular mechanisms responsible for NKG2D ligand regulation remain complex. Here we report that cancer cell metabolism supports constitutive surface expression of the NKG2D ligand MHC class I chain-related proteins A (MICA). Knockout of the *N*-glycosylation gene *N*-acetylglucosaminyltransferase V (MGAT5) in HEK293 cells induced altered metabolism and continuous high MICA surface expression. MGAT5 knockout cells were used to examine the association of cell metabolism and MICA expression through genetic, pharmacological and metabolic assays. Findings were verified in cancer cell lines. Cells with constitutive high MICA expression showed enhanced spare respiratory capacity and elevated mitochondrial efflux of citrate, determined by extracellular flux analysis and metabolomics. MICA expression was reduced by inhibitors of mitochondrial function, FCCP and etomoxir e.g., and depended on conversion of citrate to acetyl-CoA and oxaloacetate by ATP citrate lyase, which was also observed in several cancer cell types. Assay for Transposase-Accessible Chromatin using sequencing (ATAC-seq) analysis revealed that upregulated MICA transcription was associated with an open chromatin structure at the MICA transcription start site. We identify mitochondria and cytoplasmic citrate as key regulators of constitutive MICA expression and we propose that metabolic reprogramming of certain cancer cells facilitates MICA expression and NKG2D-mediated immune recognition.

## Graphical Abstract

MGAT5 knockout (KO) in HEK293 cells induces metabolic changes resulting in increased intracellular UDP-GlcNAc, increased glycolysis, enhanced spare respiratory capacity and higher citrate flux from the mitochondria. MGAT5 KO cells express constitutively high MICA, mainly regulated on the transcriptional level through opening of the chromatin at the MICA promoter. MICA expression in MGAT5 KO cells is dependent on citrate turnover and histone acetylation. Blocking citrate flux inhibits MICA expression in numerous cancer cell lines, and we propose that this is a central metabolic regulation of MICA and immune surveillance.

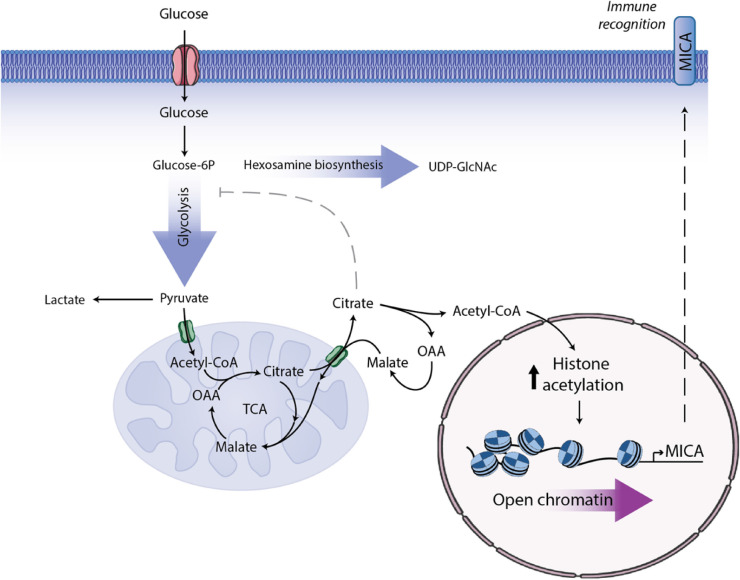

## Introduction

Natural killer (NK) and CD8^+^ T cells monitor autologous cells for markers of tumorigenesis and stress. These immune cells express the NKG2D receptor that recognizes NKG2D ligands (NKG2DLs) upregulated on the surface of transformed cells (1). NKG2DL expression is in many ways a double-edged sword. Upregulation of NKG2DLs on cancer cells enhance NK cell infiltration and promote cancer cytotoxicity (2, 3). Conversely, numerous cancer cells maintain chronic NKG2DL expression and evade immune elimination by down-modulating and impairing NKG2D receptor signaling (4–7).

Cancer cells that block NKG2DL surface expression to evade immune recognition and clearance, can be treated with stress-inducers such as histone deacetylase inhibitors (HDACi’s), heat-shock or short-chain fatty acids (SCFAs) that upregulate NKG2DLs (8). To date, studies have primarily focused on delineating transient NKG2DL induction whereas not much is known about regulation of their constitutive expression.

Metabolic reprogramming is a central hallmark of cancer. Cancer cells use aerobic glycolysis that was initially believed to be a result of dysfunctional mitochondria (9); however, later advances have shown that cancer cells often use aerobic glycolysis alongside mitochondrial oxidative phosphorylation (OXPHOS) (10). Mitochondria are not merely the powerhouse of the cell, but also provide metabolites for anabolic pathways necessary for cell growth. Citrate can be exported from the tricarboxylic acid (TCA) cycle for biosynthetic purposes. In the cytosol, citrate is cleaved by ATP citrate lyase (ACLY) to generate acetyl-CoA and oxaloacetate (OAA) (11, 12). Citrate is an inhibitor of glycolysis, thus to maintain high aerobic glycolysis, cancer cells require low cytoplasmic citrate (13). Moreover, conversion of citrate by ACLY is a critical regulator of gene transcription by producing acetyl-CoA for histone acetylation (14). Several of these cancer-associated metabolic properties are shared with other highly proliferating cells, such as activated T cells.

Expression of NKG2DLs is associated with hyperproliferation (8) and thus with highly active metabolism. Two studies have linked NKG2DL expression to active glycolysis (15, 16), whereas one study reports that inhibition of glycolysis increased basal NKG2DL expression in breast cancer cell lines (17, 18). These studies emphasize a link to proliferative cell metabolism, and suggest that the role of glycolysis in NKG2DL regulation is context-specific.

NKG2DLs fall into two groups, the UL-16 binding protein 1-6 (ULBP1-6) and the MHC class I chain-related proteins A and B (MICA and MICB). Surface expression of each NKG2DL is regulated individually and at all levels of protein biogenesis (8). We have previously shown that surface expression of specific MICA alleles depends on *N*-glycosylation (19, 20). *N*-acetylglucosaminyltransferase V (MGAT5) is an oncoprotein catalyzing the formation of β*-1,6-*branched *N*-glycans that promote surface retention of glycoproteins (21), but it is not known if MGAT5 regulates surface expression of MICA. Growth factor receptors are examples of MGAT5 substrates, and MGAT5 overexpression is associated with growth, adhesion, invasion and metastasis of cancer (22–26). Inhibition of MGAT5 reduces tumor growth, enhances the anti-tumor responses by CD4^+^ T cells and macrophages, and promotes Th1 differentiation (27, 28).

In this study we examine the metabolic regulation of the NKG2DL MICA. We discover that MICA was increased after MGAT5 knockout (KO) in a metabolically dependent way, and use this as a model to investigate the regulatory mechanisms of constitutive MICA expression. We find that glycolysis and mitochondrial export of citrate promotes constitutive MICA transcription in MGAT5 KO cells, a regulation that was also shown in several MICA-expressing cancer cells. In particular, increased MICA transcription was associated with altered chromatin accessibility of the MICA promoter. Our findings suggest that citrate drives a metabolic stress that modulates chromatin accessibility to facilitate basal MICA transcription and thereby regulate immune surveillance.

## Materials and Methods

### Animals

Female NMRI mice 6 to 10-weeks old (Taconic, Lille Skensved, Denmark) were used, and all studies were performed in accordance with the Danish Act on Animal Experimentation, which implements Directive 2010/63/EU on the protection of animals in scientific research. The studies were approved by the Animal Experimentation Inspectorate, Ministry of Environment and Food, Denmark (License No. 2017-15-0201-01262). Health monitoring was carried out in accordance with Federation for Laboratory Animal Science Associations guidelines.

### Reagents, Pharmacological Inhibitors, and DNA Constructs

Pharmacological compounds from Sigma-Aldrich were *N*-acetyl-D-glucosamine (GlcNAc, A3286), PUGNAc (A7229), 2-deoxy-D-glucose (2DG, D6134), carbonyl cyanide 4-(trifluoromethoxy)phenylhydrazone (FCCP, C2920), UK-5099 (PZ0160), bis-2-(5-phenylacetamido-1,3,4-thiadiazol-2-yl)ethyl sulfide (BPTES, SML0601), potassium hydroxycitrate tribasic monohydrate (HC, 59847), sodium dihydrogencitrate (234265), sodium acetate (S5636), oxaloacetic acid (OAA, O4126), 6-Mercaptopurine monohydrate (6-MP, 852678), azaserine (A4142), 5-aminoimidazole-4-carboxamide ribonucleotide (AICA-R, A9978), *N*-Acetylcysteine (NAc, A9165), sodium propionate (P1880), sodium butyrate (B5887), DMSO (D2438), PBS (D8537). Etomoxir sodium salt was purchased from Cayman Chemicals (Ann Arbor, MI, United States, 828934-41-4). BMS303141 was from Tocris Bioscience (Bristol, United Kingdom, 4609).

The GFP-Myc-MICA^∗^018 and MICA^∗^008 vectors, containing the coding sequences of MICA^∗^018 or MICA^∗^008 allele downstream of a generic leader, a GFP cassette, and a myc tag, were provided by Dr. M. Wills (University of Cambridge, Cambridge, United Kingdom) (29). pGL3-Basic (pGL3B) luciferase vector was purchased from Promega (Promega, Madison, WI, United States, E1751). MICA-*firefly* luciferase promoter vectors and SV40-*renilla* luciferase promoter vector were provided by Prof. C. O’Callaghan (University of Oxford, Oxford, United Kingdom) (30).

### Purification of Peripheral Blood Lymphocytes

Human peripheral blood mononuclear cells (PBMCs) were isolated by Histopaque-1077 (Sigma-Aldrich, St. Louis, MO, United States, 10771) separation from buffy coats obtained from healthy blood donors (The Capital Region Blood Bank, Copenhagen University Hospital, Copenhagen, Denmark). To obtain peripheral blood lymphocytes (PBLs), PBMCs were depleted from monocytes by incubation with Dynabeads (Invitrogen, Carlsbad, CA, United States, 11041), as previously described (31). PBLs were activated in RPMI1640 without glucose (Gibco, Gaithersburg, MD, United States, 11879-020) supplemented with 10% dialyzed fetal bovine serum (FBS) (F9665), 2 mM penicillin/streptomycin (P4333), 2 mM L-Glutamine (G7513), 1 mM sodium pyruvate (S8636) and either 10 mM D-glucose (G8769) or 10 mM D-galactose (G6404), all purchased from Sigma-Aldrich. PBLs were activated with CD3/CD28 beads (Invitrogen, 11132D) and 20U/mL hIL-2 (Peprotech, Rocky Hill, NJ, United States, 200-02) for 3 days. On day 3, PBLs were treated with 20 ng/mL FR901228 (National Cancer Institute, Bethesda, MD, United States) for 18 h.

### Cell Line Cultivation and Proliferation

Human embryonic kidney-derived HEK293 cells, the prostate cancer cell line PC-3 and the keratinocyte-derived cell line HaCaT were purchased from American Type Culture Collection (ATCC, Manassas, VA, United States). NKG2D reporter cell CT312 and the 2B4 parental cell line were kindly provided by Chiwen Chang, Trowsdale Lab, Cambridge University. The breast cancer cell lines MDA-MB231 and MCF-7 were provided by Dr. José Moreira (Department for Veterinary Disease, University of Copenhagen, Denmark) and Henrik Leffers (The State Hospital, Copenhagen, Denmark), respectively. The cervical cancer cell line HeLa was provided by Jesper Jurlander (The State Hospital, Copenhagen, Denmark). The melanoma cells SK-MEL28, FM55m1, FM78 and FM86, and the human colon adenocarcinoma cell lines HT29 and SW480 were provided by Dr. Per thor Straten (Herlev University Hospital, Denmark). HEK293, MDA-MB231 and MCF-7 cells were cultured in DMEM with GlutaMAX (Gibco, 31966047). HeLa, HaCaT, PC-3, FM55m1, FM78, FM86, SK-MEL28, and SW480 were cultured in RPMI1640 (Sigma-Aldrich, R5886), and HT29 were cultured in McCoy’s 5A medium (Sigma-Aldrich, M8403). Media were supplemented with 10% FBS and 2 mM penicillin/streptomycin. 2 mM L-Glutamine was added to RPMI1640 and McCoy’s 5A. For long-term cell culture in glucose/galactose, cells were cultured in DMEM medium without glucose (Gibco, 11966025), supplemented with 10% dialyzed FBS, 2 mM penicillin/streptomycin, 1 mM sodium pyruvate, and 10 mM glucose/galactose. All cells were kept at culture conditions 37°C and 5% CO_2_ and were passaged every 2–3 days.

For proliferation assay, WT and MGAT5 KO cells were seeded in 1 × 10^5^ or 2 × 10^5^ cells/well. For each experiment, cells were counted in triplicate wells after 24 and 48 h using the Bio-Rad TC20 automated cell counter (Bio-Rad, Hercules, CA, United States).

### Gene Editing

MGAT5 KO cells were generated by zinc finger nuclease targeting in HEK293 cells and subsequent cloning and selection was performed as described previously (32, 33). HEK293 cells were transfected with mRNA (Sigma-Aldrich) or 5 μg of endotoxin free plasmid DNA using nucleofection on an Amaxa Nucleofector (Lonza, Copenhagen, Denmark). MGAT5 KO clones were selected by loss of reactivity with L-PHA, and clones were confirmed to have Mgat5 mutations using PCR and sequencing.

Lentiviral-mediated gene transfer was performed with an MGAT5 encoding vector constructed by inserting the MGAT5 sequence (generated as a blunt-end PCR product from a vector from HW, University of Copenhagen, Copenhagen, Denmark) into an entry vector system using the pENTR Directional TOPO Cloning Kit (Invitrogen, K2435-20/K3500-20) following manufacturer’s protocol. TOPO clonal reaction entry vectors were transformed into Mach-T1 chemically competent *E. coli* using heat-shock and S.O.C. medium followed by selection. PCR inserts were confirmed by sequencing at Eurofins MWG Operons (Luxembourg). Colonies were amplified and plasmids were purified with Nucleobond Xtra Midi kit (Macherey-Nagel, Duren, Germany, 740410). MGAT5 sequences were inserted into pLX302 lentiviral destination vector with LR CLonase II enzyme mix (Invitrogen, 11789). After proteinase K treatment constructs were transformed into DH5α using heat-shock and S.O.C. medium. Selected clones were amplified and DNA was purified using Nucleobond Xtra Midi kit. Destination vectors were checked for insertion using *Bsr*GI digestion at 55°C. MGAT5-coding lentiviral particles were packaged in HEK293T cells transfected with a mix of 4 μg psPAX2 vector (packaging vector), 2 μg pCMV-VSVG (envelope vector), 5 μg pLX302 vector carrying MGAT5, and 25 μl CaCl_2_ to a final volume of 250 μl. The DNA mixture was complexed with 250 μL 2x HBS under constant air flow, and the transfection mix was added dropwise to 2 × 10^6^ HEK293T cells in antibiotic-free medium. Cell culture medium was harvested 4 days after transfection and viral particle preparations were prepared by centrifugation at 5000 × *g* for 5 min. Lentiviral particles were added to cells and incubated for 24 h. Cells were cultivated in puromycin (1 μg/mL) selection medium for 2 weeks. Functional MGAT5 expression was validated by L-PHA binding.

### Transient Transfection

Transient transfections were performed as described previously, using Amaxa Nucleofector device (Lonza) (34). DNA was introduced to 2 × 10^6^ cells in 100 μl Nucleofector solution V (Lonza, VCA-1003) and pulsed using the nucleofector program Q-001. For GFP-myc-tagged MICA^∗^008 and MICA^∗^018 constructs, cells were transfected with 1 μg DNA and analyzed the next day. Transfection with shRNAs or luciferase promoter constructs was carried out by calcium-phosphate transfection. Briefly, DNA/RNA were prepared in 10 μl CaCl_2_ (2.5M) and adjusted to a final volume of 100 μl. DNA mixture was complexed with 100 μl 2x HBS (HEPES, NaCl, Na_2_HPO_4_) and added dropwise to 2 × 10^6^ cells. Scrambled siRNA control, siIDH1 and siIDH2 ON-TARGET plus SMART pools were purchased from GE Healthcare Dharmacon (Lafayette, CO, United States).

### Functional Assays

For NKG2D down-modulation, PBLs were isolated as described above, followed by depletion of CD4^+^ cells using CD4 antibody (eBioscience, San Diego, CA, United States, 16-0049) and Dynabeads Mouse pan-IgG (Invitrogen, 11041). CD4^+^-depleted PBLs were cultured in RPMI1640 (Sigma-Aldrich, R5886) supplemented with 10% human serum (Sigma-Aldrich, H3667), 2 mM penicillin/streptomycin, 2 mM L-Glutamine and 10 ng/mL hIL-15 (Peprotech, 200-15) for 3 days to enrich for NK/CD8^+^ T cells. NKG2D down-modulation assay was performed as previously described (35). NKG2D ligands on effector cells (HEK293 WT or MGAT5 KO cells) were incubated with blocking NKG2D-Fc (R&D Systems, Minneapolis, MN, United States, 1299-NK) or control IgG1-Fc (R&D Systems, 110-HG) 8 μg/mL for 30 min at 4°C. Effector cells and target cells (NK/CD8^+^ T cells) were mixed at indicated effector:target ratios and spun down 2 min 90 × *g* to allow conjugate formation. After 2 h co-cultivation, NK/CD8^+^ T cells were analyzed for NKG2D surface expression by flow cytometry using Accuri C6 flow cytometer (BD Bioscience, Franklin Lakes, NJ, United States).

For the reporter cell assay, the NKG2D-reporter cell line 2B4-CT312 and the parental control 2B4 cell line (target cells) (36) were mixed with effector cells (WT or MGAT5 KO cells) that were either blocked with NKG2D-Fc or control IgG1-Fc as described above. Effector and target cells were co-cultivated at different E:T ratios for 14–16 h. GFP expression of target cells was assessed with Accuri C6 flow cytometer. For *in vivo* assay, target cells were labeled with Vybrant DiD cell-labeling solution (Invitrogen, V22887) according to manufacturer’s protocol, and injected intraperitoneally together with WT or MGAT5 KO cells in a 1:1 ratio, 20 × 10^6^ of each. 4–6 mice were used per group. Target cells were harvested after approximately 18 h with peritoneal lavage and NKG2D activation of DiD-positive reporter cells were assessed as GFP expression with Accuri C6 flow cytometer.

### Lactate and dNTP Measurements

Concentrations of L-Lactate was measured enzymatically with Randox colorimetric assay according to manufacturer’s protocol (Randox, Crumlin, United Kingdom, LC2389). Reaction and analysis was performed on an Advia 1800 Chemistry System (Siemens, Munich, Germany).

dNTP levels were determined in 2 × 10^6^ cells harvested with trypsinization and pelleted by centrifugation for 1200 × *g* for 5 min followed by resuspension of cell pellets in 60% methanol, frozen in liquid nitrogen and boiled at 100°C for 3 min. Samples were evaporated until dryness in a speedvac and whole cell levels of dTTP dATP, dCTP, and dGTP were determined using the DNA polymerase assay previously described (37).

### LC-HRMS Metabolite Profiling

To determine intracellular metabolite levels, cell pellets from 1 × 10^6^ cells were resuspended in 750 μl of cold methanol after 5 min sonication. Samples were prepared by 15 s vortex followed by 15 min equilibration at room temperature. After centrifugation at 13000 × *g* for 10 min at 4°C, 400 μl supernatants were collected, transferred to Ultrafree-MC Centrifugal filter devices (Merck Millipore Ltd., Cork, Ireland), and centrifuged at 10000 × *g* for 4 min at 4°C. From this, 100 μl was transferred to LC vials, and 30 μl of each sample was pooled to a mixed QC sample.

LC-HRMS was performed on a 1290 Infinity II ultra-high performance liquid chromatography (UHPLC) system coupled to a 6550 iFunnel quadrupole-time of flight (Q-TOF) mass spectrometer equipped with a dual AJS electrospray ionization source (Agilent Technologies, Santa Clara, CA, United States). Polar metabolites were separated on a SeQuant ZIC-HILIC (Merck, Darmstadt, Germany) column 100 Å (100 mm × 2.1 mm, 3.5 μm particle size) coupled to a guard column (20 mm × 2.1 mm, 3.5 μm particle size) and an inline-filter. Mobile phases consisted of 0.1% formic acid in water with (solvent A) and 0.1% formic acid in acetonitrile with (solvent B). The elution gradient used was as follows: isocratic step at 95% B for 1.5 min, 95% B to 40% B in 12 min and maintained at 40% B for 2 min, then decreasing to 25% B at 14.2 min and maintained for 2.8 min, then returned to initial conditions over 1 min, and the column was equilibrated at initial conditions for 7 min. The flow rate was 0.3 mL/min, injection volume was 2 μL and the column oven was maintained at 25°C. The acquisition was obtained with a mass range of 50–1200 *m/z* for, where full scan high-resolution data is acquired at three alternating collision energies (0 eV, 10 eV and 30 eV). Positive and negative raw LC-HRMS files were independently processed with an in-house developed PCDL library for polar metabolites using Profinder version B.06 (Agilent Technologies). Identification of reported compounds was assessed by accurate mass and retention time (AMRT) plus fragment identification at two collision energies (10 and 30 eV). Detailed acquisition methodology has been described previously (38, 39). UDP-GlcNAc/UDP-GalNAc detected peak (screened by expected calculated mass) could be of either compound as these two sugars could not be separated chromatographically, hence has been reported as a putative metabolite pending confirmation.

### Extracellular Flux Analysis

The Seahorse XFe96 extracellular flux analyzer (Agilent Technologies) was used to measure OCR and ECAR on HEK293 cells. Cells were seeded at the density 2 × 10^4^ cells/well ∼24 h before the experiment. One hour prior to assay run, cells were rinsed and switched to XF media (Agilent Technologies) with 1 mM sodium pyruvate and 10 mM glucose or galactose and incubated at 37°C CO_2_-free incubator. For the mitochondrial stress tests, OCR was measured under basal conditions, and during sequential injection of 1 μM oligomycin (Sigma-Aldrich, 495455), 1 μM FCCP (Sigma-Aldrich, C2920), and 0.5 μM rotenone (Rot, Sigma-Aldrich, R8875) + 0.5 μM antimycin A (AA, Sigma-Aldrich, A8674). Reported basal respiration is calculated from the third measuring point with OCR after Rot and AA subtracted, ATP-coupled respiration display OCR after oligomycin subtracted from the third measuring point, and maximal respiration is OCR after FCCP with OCR after Rot and AA subtracted.

For measuring the effect of HC, OCR was assessed 2 h after an injection of 15 mM HC.

### ^13^C_6_-Glucose Tracing Experiment

1 × 10^6^ cells were incubated for 1 h in DMEM medium without glucose supplemented with 10% FBS, 1 mM sodium pyruvate, and 25 mM uniformly labeled [U-^13^C]-Glucose (Cambridge Isotope Laboratories, Tewksbury, MA, United States, CLM-1396). Incubation medium samples were collected and cleared by centrifugation 300 × *g* for 5 min. Cells were washed and detached sterically. Intracellular metabolites were extracted in 70% ethanol, and centrifuged at 20.000 × *g* for 20 min (4°C) to separate the soluble extract (supernatant) from the insoluble components (pellet). Cell extracts and medium samples were lyophilized and reconstituted in water for subsequent biochemical analyses. Extract samples were adjusted to pH 1-2 with HCl and evaporated to dryness under nitrogen flow. Analytes were extracted into an organic phase (96% ethanol/benzene) followed by derivatization with 14% DMF/86% MTBSTFA with a modified procedure from (40). Standards containing unlabeled metabolites of interest and cell extracts were separated and analyzed in a gas chromatograph (Agilent Technologies 7820A chromatograph, J&W GC column HP-5MS, parts no. 19091S-433) coupled to a mass spectrometer (Agilent Technologies, 5977E). The isotopic enrichment of the metabolites of interest was corrected for natural abundance of ^13^C using the unlabeled standards and calculated according to (41). Data are presented as labeling (%) of M + X, where M is the mass of the unlabeled molecule and X is the number of labeled C-atoms in a given metabolite (42).

### Western Blotting

Proteins were extracted using RIPA buffer (Thermo Scientific, Waltham, MA, United States, 89901) and proteinase/phosphatase inhibitor cocktail (Thermo Scientific, 1861281) for 30 min on ice. Lysates were sonicated 2 times for 15 s and cleared by centrifugation at 13,000 rpm for 10 min at 4°C. Protein extracts were denatured at 70°C for 10 min in NuPAGE sample buffer and DTT (Sigma-Aldrich, 646563). Proteins were resolved using 4–12% SDS-PAGE gels (Invitrogen) and transferred to nitrocellulose membranes (Invitrogen, IB301001) using the iBlot device (Invitrogen). For total protein stain, membranes were washed in ddH2O and stained with REVERT protein stain solution (LI-COR Biosciences, Lincoln, NE, United States, 926-11015) according to manufacturer’s protocol. Membranes were blocked in TBST blocking buffer (LI-COR Biosciences, 927–50000), probed with primary antibodies in TBS w. 0.1% Tween 20 and 5% BSA overnight on a shaker at 4°C, and washed in TBS + 0.1% Tween 20. Secondary antibody was from LI-COR (LICOR Biosciences, 926–32212) and signals were visualized by the Odyssey Fc Imaging System (LI-COR Biosciences). *O*-GlcNacylation was detected with RL2 *O*-GlcNAcylation antibody (Abcam, Cambridge, United Kingdom, ab2739), ATP citrate lyase (ACLY) was detected with rabbit ACLY antibody (Cell Signaling; 4332), and ACLY phosphorylation with rabbit phospho-ACLY (Ser455) antibody (Cell Signaling; 4331).

### Flow Cytometry

Adherent cells were detached in PBS w. 1 mM EDTA (Invitrogen, 15575-038) or by pipetting. Cell surface staining was done as previously described (43), and cells were analyzed on Accuri C6 flow cytometer (BD Bioscience). Antibodies used for this study were: MICA (R&D systems, FAB1300A), ULBP2/5/6 (R&D Systems, FAB1298P), NKG2D (R&D Systems, FAB139A), ULBP1 (R&D Systems, FAB1380P), ULPB3 (R&D Systems, FAB1517A), ULBP4 (R&D Systems, FAB6285A), MICA/B (BD Bioscience, 558352), ICAM-1 (Leinco Technologies, C170), mouse IgG1 anti-myc-tag (Merck Millipore, 05724), MICB (R&D Systems; MAB1599) or IgG2b isotype control (R&D Systems; MAB004) detected with secondary anti-mouse IgG (Biolegend, San Diego, CA, United States, 405308). Binding of fluorescently labeled AF647-L-PHA (Invitrogen, L32457) and FITC-E-PHA (Vector Laboratories, Burlingame, CA, United States, FL-1121) was used to measure surface levels of complex *N*-glycans. All isotype controls were purchased from BD Bioscience.

For staining with mitochondrial probes, neutral lipid stains or 2-NBDG uptake, 5 × 10^5^ cells seeded the day prior to experiment were washed once in PBS and incubated for 15 min at 37°C and 5% CO_2_ in warm growth medium containing 25 nM tetramethylrhodamine methyl ester perchlorate (TMRM, Sigma-Aldrich, T5428), 10 nM MitoTracker Green FM (Invitrogen, M7514), or for 2 h in growth medium with 5 μM 2-NBDG (Invitrogen, N13195). Bodipy 493/503 (Invitrogen, D3922) was diluted in warm serum-free medium in a 1:7000 dilution and shaken vigorously to solubilize the lipids, immediately before loading into the cells for 15 min. Cells were washed twice in PBS + 2% FBS and detached sterically prior to analysis.

The soluble NKG2D–Fc receptor (1299-NK, R&D Systems) and IgG1–Fc (110-HG; R&D Systems) were labeled with Zenon Alexa Fluor 647 against human IgG1 (Z25408; Invitrogen) prior to staining of melanoma cells.

Data were acquired with an Accuri C6 instrument using Accuri C6 software, and analyzed in Flowlogic v7.2.1 (Inivai Technologies, Mentone, VIC, Australia) by gating on viable cells in forward-side-scatter plots (FSC/SSC) followed by single cell gating by area-height-scatter plots (FSC-A/FSC-H). Geometric mean fluorescent intensity (MFI) values are displayed in figures as MFI, or with corresponding isotype control subtracted as ΔMFI.

### Real Time PCR Analysis

Total RNA was extracted by phase separation in TRIzol-chlorophorm and purified on Direct-zol spin-columns (Zymo Research, Irvine, CA, United States) according to manufacturer’s protocol. cDNA was generated using SuperScript cDNA synthesis kit (Invitrogen) under standard PCR conditions. Following primer sequences were used for quantitative RT-PCR with Brilliant SYBR Green qPCR Master Mix Kit: MICA (MICA_F: TGGCAGACATTCCATGTTTCTG, MICA_R: CTCGTCCCAACTGGGTGTTG), ULBP2 (ULBP 2_F: CAGAGCAACTGCGTGACATT, ULBP2_R: GGCCAC AACCTTGTCATTCT), IDH1 (IDH1_F: CTATGATGGTGA CGTGCAGTCG, IDH1_R: CCTCTGCTTCTACTGTCTTGCC), IDH2 (IDH2_F: AGATGGCAGTGGTGTCAAGGAG, IDH 2_R: CTGGATGGCATACTGGAAGCAG), GLUT1 (GLUT1_F: CTGCTCATCAACCGCAAC, GLUT1_R: CTTCTTCTCCCG CATCATCT), GLUT2 (GLUT2_F: TACATTGCGGACTTCTG TGG, GLUT2_R: AGACTTTCCTTTGGTTTCTGG), GLUT3 (GLUT3_F: CAGCGAGACCCAGAGATG, GLUT3_R: TTGG AAAGAGCCGATTGTAG), GLUT4 (GLUT4_F: TGGGCTT CTTCATCTTCACC, GLUT4_R: GTGCTGGGTTTCACCTC CT), and RPLP0 as housekeeping gene (RPLP0_F: CCTCGTGGAAGTGACATCGT, RPLP0_R: CATTCCCCC GGATATGAGGC). Real-time qPCR was performed on Bio-Rad CFX96 Real-time Thermal Cycler C1000 Touch, and all transcripts were normalized to housekeeping RPLP0 transcript.

### Luciferase Reporter Assay

Cells were transiently transfected, using calcium-phosphate transfection as described above, with *firefly* luciferase promoter vectors (1 μg) and an SV40-promoter driven *renilla* luciferase vector (0.5 μg). Cells were harvested and snap frozen 24 h post transfection. Pellets were lysed in Dual-Glo Luciferase Reagent (Promega, E2920) and *firefly* luciferase activity was analyzed by luminometer Microbeta II (PerkinElmer, Waltham, MA, United States). *Renilla* luciferase activity was recorded by the instrument after subsequent addition of 1:1 volume Dual-Glo Stop & Glo (Promega, E2920). To correct for transfection efficiency, *firefly* luciferase signals were normalized to SV40 *renilla* luciferase signals of corresponding sample.

### ATAC-Seq

ATAC-seq was performed as described previously (44). For each cell line, 50,000 cells were harvested from 3 separate cultures and used to prepare tagmented chromatin (3 replicates of WT and 3 replicates of MGAT5 KO cell lines, 6 samples total). Quality of PCR-amplified sequencing libraries was assessed using a Tapestation 2200 instrument with high sensitivity DNA screentapes (Agilent). Libraries were sequenced as paired end reads on a single lane of an Illumina HiSeq4000 flow cell. Resulting reads were aligned to the GRCh37/hg19 reference genome using Rsubread (45), and alignments were filtered to remove low quality, duplicate, and mitochondrial reads. Peaks were called using MACS2 (46) on merged reads from all samples, and differential peak accessibility between cell lines was determined using edgeR (47) with a threshold false discovery rate of 5%. Transcription factor binding motifs enriched in differentially accessible peaks were identified using HOMER (48). H3K4me3 ChIP-seq data were downloaded from ENCODE^1^ and are available under accession ENCFF756EHF.

### Quantification and Statistical Analysis

Results are presented as mean ± SEM. Differences were analyzed for statistical significance using Prism 7 or 8 (GraphPad Software, La Jolla, CA, United States). Statistical analysis was performed as stated in figure legends, using unpaired *t*-test in 1A, 1C, 1E, 3E-F, 3H, 5C, 7A, 7E-F, paired *t*-test in 4F-G, 7D, multiple *t*-test in 1B, 1D, 3D, 4A-B, one sample *t*-test in 2A-C, 3C, 4C, 4E, 7G, two-way ANOVA in 3A, 5D-F, 5H-I, 6A, 6E, 7h-I, or one-way ANOVA in 5G. Level of statistical significance was determined by ^∗^*p* < 0.05, ^∗∗^*p* < 0.01 and ^∗∗∗^*p* < 0.001, ^****^p < 0.0001.

## Results

### MGAT5 Knockout Increases NKG2DL Expression and Activates NKG2D *in vitro* and *in vivo*

**FIGURE 1:**
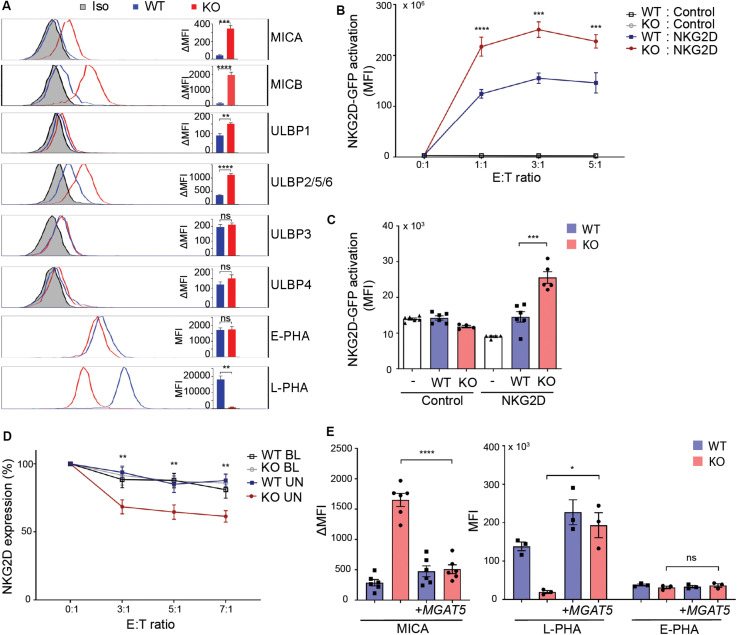
MGAT5 knockout increases NKG2DL expression and activates NKG2D *in vitro* and *in vivo*. **(A)** Surface expression of NKG2D ligands and binding of fluorescently labeled L-PHA (MGAT5 modifications) or E-PHA (MGAT3 modifications) on HEK293 wildtype (WT) and HEK293 MGAT5 knockout (KO) cells or isotype control staining (Iso) analyzed by flow cytometry. Data are presented as histograms representative of at least three independent experiments and in bar graphs showing mean fluorescence intensity (MFI). **(B)** *In vitro* NKG2D activation measured as GFP expression in NKG2D negative reporter cells (Control) and NKG2D expressing (NKG2D) reporter cells (target cells) co-cultivated with WT or KO cells (effector cells) for 14–16 h at indicated effector:target (E:T) ratios. **(C)** NKG2D activation *in vivo* measured on reporter cells as in (B) after activation by WT or KO at a 1:1 ratio in peritoneum of NMRI mice for approximately 18 h. GFP expression in DiD-labeled reporter cells signifies NKG2D activation and is shown as GFP MFI values of cells from four-six mice per group. **(D)** NKG2D down-modulation was assessed on NK/CD8^+^ T cells (target cells) after co-cultivation for 2 h with WT or KO cells (effector cells) at indicated effector:target ratios (E:T). NKG2DLs on target cells were blocked with NKG2D-Fc (BL) or unblocked with IgG1-Fc (UN). The graph depicts surface expression of NKG2D presented relative to surface NKG2D expression on target cells alone. **(E)** MICA surface expression (left) and L-PHA/E-PHA surface binding (right) after lentiviral introduction of MGAT5 into WT or KO cells. MFI values from antibody staining were corrected for isotype background staining (ΔMFI). Statistical analysis was performed by unpaired *t*-tests in **(A,C,E)**, and multiple *t*-test with 5% FDR comparing WT and KO in **(B,D)**. *p < 0.05, **p < 0.01, ***p < 0.001, and ****p < 0.0001.

Regulation of constitutive MICA expression remains largely unknown. Surface expression of certain MICA alleles depends on *N*-linked glycosylation (19, 20, 49). We questioned whether the cancer-associated glycosyltransferase MGAT5 is required for MICA expression. To assess the role of MGAT5 in regulation of NKG2DL surface expression, MGAT5 KO clones were generated in HEK293 cells. Remarkably, MGAT5 KO resulted in a permanently increased surface expression of the NKG2DLs MICA, MICB, and ULBP2/5/6 compared with parental wildtype (WT) cells (Figure 1A). To confirm MGAT5 KO we measured binding of Leukoagglutinin from *P. vulgaris* (L-PHA) that binds specifically to MGAT5-modified *N*-glycans. As expected, L-PHA binding was reduced, whereas binding of Erythroagglutinin from *P. vulgaris* (E-PHA) that interacts with MGAT3-modified *N*-glycans, was unaffected, thus verifying functional knockout of MGAT5 (Figure 1A). Modification of MGAT5 expression therefore associated with substantial changes in constitutive expression of several NKG2DLs.

To verify the functionality of MGAT5 KO-induced NKG2DLs, we tested NKG2D activation in a reporter cell line expressing human NKG2D coupled to DAP10-CD3ζ signaling and Nuclear factor of activated T cells (NFAT)-controlled GFP, ultimately expressing GFP in response to NKG2D activation (36). NKG2D-GFP activation was higher after co-cultivation with MGAT5 KO cells than with WT cells (Figure 1B), corresponding to the increased NKG2DL expression in MGAT5 KO cells (Figure 1A). The reporter cells without NKG2D (Supplementary Figure S1A) remained inactivated, indicating that the activation was NKG2D-mediated (Figure 1B). Moreover, blocking NKG2DLs with soluble NKG2D-Fc receptor impaired the activation, further validating NKG2D specificity (Supplementary Figure S1B).

To test if MGAT5 KO cells could activate NKG2D *in vivo*, we adoptively injected NKG2D reporter cells together with WT or MGAT5 KO cells into the peritoneum of NMRI mice and measured GFP expression in reporter cells. In line with our *in vitro* data, we observed a significant increase in NKG2D-GFP activation by MGAT5 KO cells compared with WT cells. The response was NKG2D-specific since the control reporter cells were unaffected (Figure 1C). These data verify that MGAT5 KO-induced NKG2DLs maintain their functional integrity *in vivo*.

NKG2D is down-modulated upon activation (50). To further examine the functionality of NKG2DL expression caused by MGAT5 KO, we assessed NKG2D downregulation after receptor activation. NKG2D was further downregulated on CD4^+^-depleted peripheral blood lymphocytes (PBLs) after co-cultivation with MGAT5 KO cells than with WT cells, and this downregulation was abolished by blocking NKG2DLs with a soluble NKG2D-Fc receptor (Figure 1D). Combined, these data indicate that KO of MGAT5 upregulates MICA and ULBP2/5/6, resulting in NKG2D activation *in vitro* and *in vivo*.

To ensure that the MICA upregulation was a result of MGAT5 KO, we stably transfected MGAT5 into WT and MGAT5 KO cells. L-PHA binding was restored within days after transfection, confirming expression of functional MGAT5. Interestingly, it took multiple passages for MICA expression to decrease to WT levels (Figure 1E and Supplementary Figure S1C), suggesting that MICA is regulated in response to a long-term adaptation to altered MGAT5 expression.

### UDP-GlcNAc Upregulates MICA Expression

**FIGURE 2:**
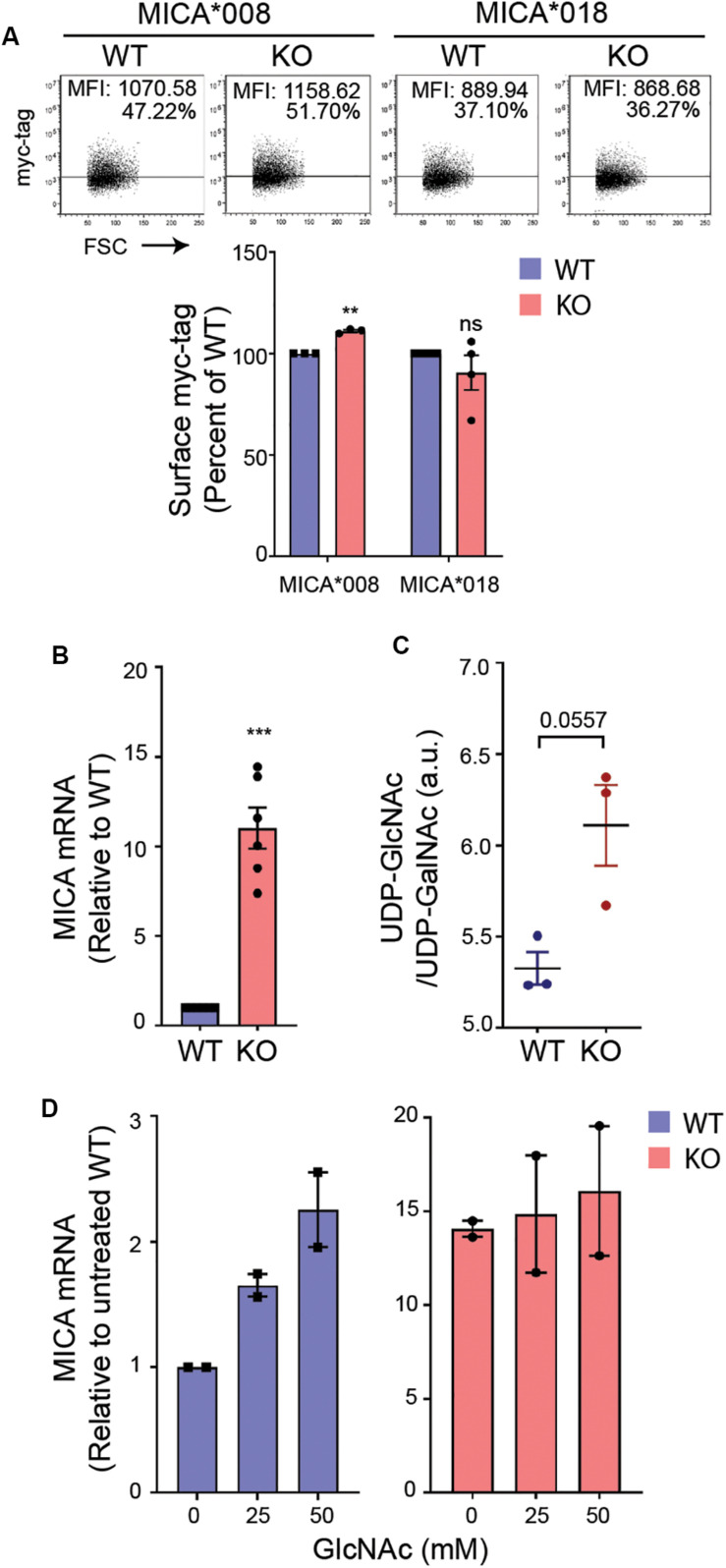
UDP-GlcNAc upregulates MICA expression. **(A)** HEK293 wildtype (WT) and HEK293 MGAT5 knockout (KO) cells were transfected with GFP-myc-tagged MICA*008 or MICA*018 under CMV promoter and detected on the surface the following day as surface myc-tag expression on GFP-positive cells. Data are displayed as isotype-corrected mean fluorescence intensity (ΔMFI) relative to WT from three-four independent experiments. Dot plots are representative of all experiments. Grid is set to 5% of corresponding isotype controls. **(B)** MICA mRNAs were analyzed by quantitative RT-PCR in total RNA purified from WT and KO cells. MICA expression was normalized to housekeeping gene RPLP0 and shown as ratio relative to WT cells from six independent experiments. **(C)** UDP-GlcNAc/UDP-GalNAc measurements from LC-HRMS analysis of intracellular metabolites in WT and KO cells are presented as relative peak areas (a.u.) corresponding to UDP-GlcNAc or UDP-GalNAc. Log_10_-transformed values from three experiments are shown as individual dots. **(D)** MICA mRNA analyzed by quantitative RT-PCR in total RNA purified from WT and KO cells after 8 days cultivation with indicated concentrations of GlcNAc. MICA expression is normalized to housekeeping gene RPLP0 and displayed as ratio relative to untreated WT cells from two independent experiments. All data are displayed as mean ± SEM. Statistical analysis was performed using one-sample *t*-test in **(A,B)**, and unpaired *t*-test with Welch’s correction in **(C)**. **p < 0.01 and ***p < 0.001, *p*-value is presented in **(C)**.

Long-term MGAT5 deficiency will likely result in aberrant *N*-glycosylation and an accumulation of the MGAT5 donor substrate UDP-*N*-acetylglucosamine (UDP-GlcNAc). To address if MICA was regulated by a change in *N*-glycosylation in MGAT5 KO cells, we assessed the post-translational regulation of MICA by measuring surface expression of transgenically expressed GFP-myc-tagged MICA under a cytomegalovirus (CMV) promoter. The MICA alleles, MICA^∗^008 and MICA^∗^018 are distinctly regulated posttranslationally (19), and although MICA^∗^008 was upregulated in MGAT5 KO cells, the regulation was minor and unlikely to account for the profound change in endogenously expressed MICA (Figures 1A, 2A). MICA transcripts on the other hand, were highly increased in MGAT5 KO cells (Figure 2B), as well as ULBP2 mRNA (Supplementary Figure S2A), suggesting that NKG2DLs are transcriptionally regulated in MGAT5 KO cells. Notably, we found that the MGAT5 substrate UDP-GlcNAc, although indistinguishable from UDP-*N*-acetylgalactosamine (UDP-GalNAc) tended to be higher in MGAT5 KO cells compared with WT cells (Figure 2C). This prompted us to address whether UDP-GlcNAc accumulation increased MICA mRNA. We found that cultivation of cells in GlcNAc-supplemented medium, which is known to increase intracellular UDP-GlcNAc through the salvage pathway (51), increased MICA transcripts and MICA surface expression, particularly in WT cells (Figure 2D and Supplementary Figure S2B). Together, this suggests that MICA is upregulated due to UDP-GlcNAc accumulation in MGAT5 KO cells by transcriptional regulation, and thus independently of MICA *N*-glycosylation.

### Glycolysis Supports MICA Expression in MGAT5 KO Cells

Accumulation of UDP-GlcNAc can regulate gene transcription directly as the donor substrate for *O*-GlcNAcylation, which competes with phosphorylation of Ser/Thr on several enzymes and transcription factors (52, 53). Moreover, UDP-GlcNAc is synthesized from the hexosamine biosynthetic pathway arising from glycolysis, and can regulate cell metabolism through *N*-glycosylation of nutrient transporters and receptors (26).

**FIGURE 3:**
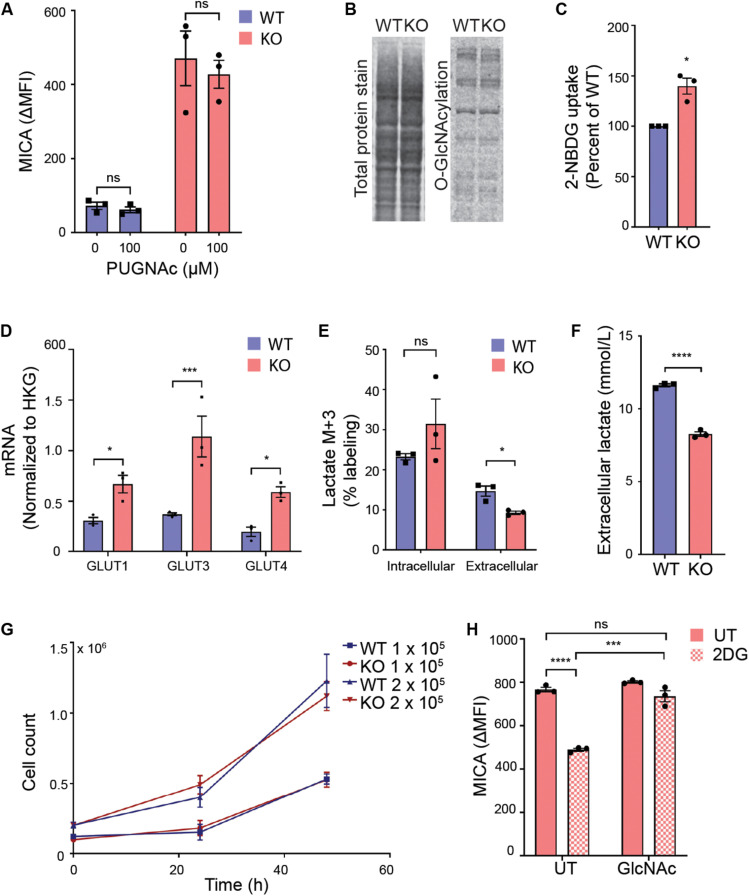
Glycolysis supports MICA expression in MGAT5 KO cells. **(A)** HEK293 wildtype (WT) and HEK293 MGAT5 knockout (KO) cells were treated with GlcNAcase inhibitor PUGNAc for 18 h prior to flow cytometry analysis of MICA surface expression. **(B)** O-GlcNAcylation of whole cell proteins from WT and KO cells quantified by western blotting of O-GlcNAcylation with the monoclonal RL2 antibody and total protein stain. Data are representative of three independent experiments. **(C)** Uptake of fluorescent glucose analog 2-NBDG in WT and KO cells after 2 h incubation with medium containing 2-NBDG (5 μM) was analyzed by flow cytometry. Mean fluorescent intensity (MFI) values are displayed as percentage of WT. **(D)** mRNA of glucose transporters GLUT1, 3, and 4 analyzed by quantitative RT-PCR in total RNA purified from WT and KO cells are presented as mRNA relative to housekeeping gene (HKG) RPLP0. **(E)** Intracellular and extracellular glucose-derived lactate in WT and KO cells were quantified by GC mass spectrometry after 1 h incubation in culture medium containing ^13^C_6_-Glucose. ^13^C incorporation in lactate is shown as percent lactate M + 3 out of total lactate fraction in cell lysates or supernatants. **(F)** Total extracellular lactate measurements from cell culture supernatants conditioned by WT or KO cells for 12 h. **(G)** Cell counts from start concentration of 1 × 10^5^ or 2 × 10^5^ cells/well counted after 24 and 48 h. **(H)** MICA surface expression on KO cells were treated with 2DG (20 mM) with or without GlcNAc (25 mM) for 22–24 h. Data of untreated (UT) samples share values from UT samples in Figures 7E,F. All MFI values from antibody staining were corrected for isotype background staining (ΔMFI). All graphs are displayed as mean ± SEM from three independent experiments. Statistical analysis was performed by two-way ANOVA with Sidak’s multiple comparison test in **(A)**, one-sample *t*-test in **(C)**, multiple *t*-test in **(D)**, and unpaired *t*-test with Welch’s correction used when SD was unequal in **(E,F,H)**. *p < 0.05, ***p < 0.001, and ****p < 0.0001.

We first explored if UDP-GlcNAc regulated MICA expression through *O*-GlcNAcylation. *O*-GlcNAc is added enzymatically to proteins by *O*-GlcNAc transferase (OGT) and removed by *O*-GlcNAcase. Inhibition of either enzyme is known to impair functional *O*-GlcNAcylation (54, 55). We blocked *O*-GlcNAcase with PUGNAc, and found that this had no effect on MICA expression (Figure 3A). In agreement with this, there was no increase in total protein *O*-GlcNAcylation in MGAT5 KO cells (Figure 3B). This indicates that *O*-GlcNAcylation is not a direct regulator of MICA, although altered *O*-GlcNAcylation could still be contributing to the long-term adaptation by modifying targets further upstream of MICA transcription.

Next, we approached the metabolic alterations in MGAT5 KO cells. Both MGAT5 and UDP-GlcNAc have been associated with regulation of cell metabolism (56–59). We therefore investigated whether glucose intake had changed in MGAT5 KO cells. Indeed, these cells had an increased uptake of the glucose analog 2-NBDG compared with WT cells, corresponding to a transcriptional upregulation of glucose transporters GLUT1, 3 and 4 (Figures 3C,D). Furthermore, when we traced ^13^C_6_-labeled glucose, we found that MGAT5 KO cells converted glucose into intracellular lactate; however, the glucose-derived lactate in MGAT5 KO cells was not secreted from the cells, resulting in overall lower extracellular lactate (Figures 3E,F). The increased uptake of glucose and conversion to intracellular lactate in MGAT5 KO cells resembled elevated aerobic glycolysis, which is one of the hallmarks of cancer and supports increased growth and proliferation (60). Nevertheless, we found that WT and MGAT5 KO cells proliferated at the same rate (Figure 3G). Importantly, impairing glycolysis in MGAT5 KO cells with 2-deoxy-D-glucose (2DG) reduced MICA mRNA and surface expression (Figure 3H and Supplementary Figure S3A). 2DG inhibits the flux through glycolysis as well as the hexosamine biosynthetic pathway. Addition of GlcNAc or D-mannose rescues the hexosamine biosynthetic pathway (61), and we found that it also restored MICA expression after 2DG inhibition (Figure 3H and Supplementary Figure S3B), suggesting that MICA expression in MGAT5 KO cells depends on glucose flux to the synthesis of UDP-GlcNAc.

Recently, it was shown that purine biosynthesis was critical for glucose-induced MICA expression (16). We therefore tested if MICA expression in MGAT5 KO cells was caused by enhanced purine biosynthesis, but MICA expression in MGAT5 KO cells was unaffected by the purine biosynthesis inhibitor 6-mercaptopurine (6-MP) and even slightly increased by the inhibitor azaserine (Supplementary Figures S3C,D). Moreover, the precursor for *de novo* purine biosynthesis AICA-R upregulated MICA expression in both WT and MGAT5 KO cells (Supplementary Figure S3E), further suggesting that the constitutive MICA expression on MGAT5 KO cells is not caused by consistently increased purine biosynthesis. Overall, these data indicate that glycolytic flux to the hexosamine biosynthetic pathway supports constitutively upregulated MICA expression in MGAT5 KO cells.

### MICA Expression Requires Functional Adaptation of Mitochondria in MGAT5 KO Cells

**FIGURE 4:**
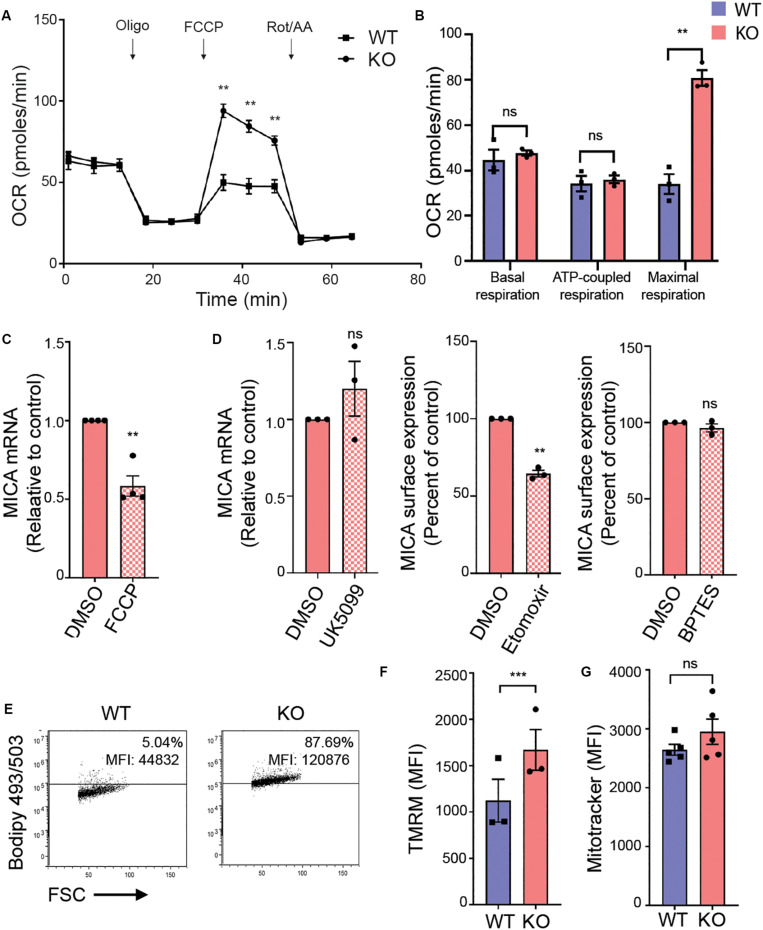
MICA expression requires functional adaptation of mitochondria in MGAT5 KO cells. **(A,B)** Mitochondrial stress test performed on HEK293 wildtype (WT) and HEK293 MGAT5 knockout (KO) cells and analyzed by Seahorse XF analysis. **(A)** Fluctuations in oxygen consumption rate (OCR) after addition of oligomycin (1 μM), FCCP (1 μM) and rotenone/antimycin A (Rot/AA) (0.5 μM). **(B)** Shows quantifications of basal respiration (third measuring point) and maximal respiration (OCR after FCCP addition) adjusted for non-mitochondrial oxygen consumption (OCR after Rot/AA), and ATP-coupled respiration (the drop in OCR after oligomycin). Data shows mean ± SEM of three independent experiments. **(C)** MICA mRNA analyzed by quantitative RT-PCR in total RNA purified from KO cells after 4 h treatment with DMSO or the mitochondrial uncoupler FCCP (2.5 μM). MICA expression is normalized to housekeeping gene RPLP0 and displayed as ratio relative to vehicle (DMSO) as mean ± SEM of four independent experiments. **(D)** MICA expression after inhibition of TCA cycle substrates: MICA mRNA analyzed by quantitative RT-PCR in total RNA purified from KO cells after 4 h treatment with DMSO or the pyruvate import inhibitor UK5099 (200 μM). MICA expression is normalized to housekeeping gene RPLP0 and displayed as ratio relative to DMSO control (left). MICA surface expression analyzed by flow cytometry in KO cells after 18 h treatment with fatty acid import inhibitor etomoxir (500 μM) (middle) or glutaminolysis inhibitor BPTES (5 μM) (right). Mean fluorescent intensity (MFI) values are corrected for isotype background staining and displayed as percent of DMSO control. All three graphs display mean ± SEM from three independent experiments. **(E)** Neutral lipid stain with Bodipy 493/503 probe was loaded to WT and KO cells and detected by flow cytometry. Grid is set at ∼5% according to stained WT cells. Dot plots are representative of four independent experiments. **(F,G)** Mitochondrial membrane potential **(F)** and mitochondrial mass **(G)** in WT and KO cells measured with the fluorescent probe TMRM (25 nM) or MitoTracker Green FM (10 nM), respectively. The bar graphs display mean ± SEM of MFI values from three-five independent experiments. Statistical analysis was performed by multiple *t*-test with a 5% false discovery rate in **(A,B)**, and one-sample *t*-test in **(C,E)**, and paired *t*-test in **(F,G)**. **p < 0.01 and ***p < 0.001.

The hexosamine biosynthetic pathway plays a key role in rewiring cancer metabolism (62). We therefore evaluated mitochondrial OXPHOS in an extracellular flux assay that monitors oxygen consumption rate (OCR) in response to specific inhibitors of oxidative metabolism (Figures 4A,B). Basal respiration and ATP-coupled respiration were unaltered in MGAT5 KO cells (Figures 4A,B). Remarkably, the maximal respiration was more than twice as high in MGAT5 KO cells compared with WT cells (Figures 4A,B), demonstrating an enhanced spare respiratory capacity (SRC). To determine if mitochondrial functionality was necessary for MICA expression, we impaired the mitochondrial membrane potential with the uncoupler FCCP, and found that it reduced MICA mRNA and cell surface expression in MGAT5 KO cells (Figure 4C and Supplementary Figure S4A). The TCA cycle can be supplied with metabolites from glycolysis, fatty acid oxidation and amino acid catabolism. Certain immunological functions have been associated with specific TCA cycle substrates (63). To test if MGAT5 KO cells supported MICA expression by oxidation of either of these resources, we inhibited the mitochondrial pyruvate carrier (MPC), import of fatty acids by carnitine palmitoyltransferase 1 (CPT1) or glutaminolysis by glutaminase (GLS) with the pharmacological inhibitors UK5099, etomoxir and BPTES, respectively (Figure 4D). The color of the MPC inhibitor obstructed flow cytometric analysis, but we found that it had no effect on MICA transcription (Figure 4D). The CPT1 inhibitor etomoxir reduced MICA transcript levels and surface expression in MGAT5 KO cells, whereas inhibition of GLS had no effect (Figure 4D and Supplementary Figure S4B). MICA mRNA levels in WT cells were not affected by etomoxir, but the response in WT cells was otherwise comparable (Supplementary Figures S4B,C). These data suggest that constitutive MICA expression in MGAT5 KO cells depends on fatty acid oxidation. Accordingly, MGAT5 KO cells also had increased accumulation of neutral lipids, providing plenty of substrate for fatty acid oxidation (Figure 4E). However, etomoxir has recently been shown to have inhibitory effects on OXPHOS independent of fatty acid oxidation, suggesting that MICA expression may be independent of fatty acid oxidation, but supporting the importance of functional mitochondria (64). We further tested if MGAT5 KO-induced MICA expression was regulated by the mitochondrial production of reactive oxygen species (ROS), but the antioxidant *N*-Acetylcysteine (NAc) potentiated rather than reduced MICA expression (Supplementary Figure S4D). Furthermore, MGAT5 KO cells had a higher membrane potential measured with the mitochondrial directed probe TMRM, but no difference in mitochondrial mass, quantified with the fluorescent probe MitoTracker (Figures 4F,G), demonstrating that the enhanced SRC was not caused by an increased mitochondrial mass.

Together, our data indicate that mitochondrial properties are altered in MGAT5 KO cells evident by an increased membrane potential and enhanced SRC, and disrupting mitochondrial integrity reduced MICA expression. Mitochondrial function is thus critical for the increased MICA expression in MGAT5 KO cells, but not by producing ROS.

### Mitochondrial Export of Citrate Facilitates MICA Expression in MGAT5 KO Cells

**FIGURE 5:**
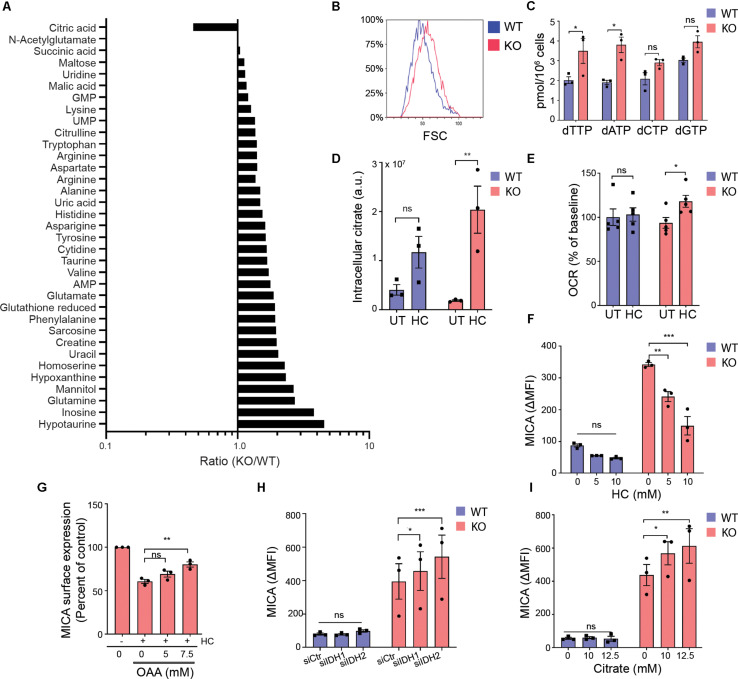
Mitochondrial export of citrate facilitates MICA expression. **(A)** Intracellular metabolites in HEK293 wildtype (WT) and HEK293 MGAT5 knockout (KO) cells quantified by LC-HRMS. Relative peak areas (a.u.) of identified metabolites (N:35) from three experiments are pooled, expressed as ratios (KO/WT) and sorted accordingly. **(B)** Cell size of WT and KO cells measured as forward scatter (FSC) by flow cytometry, representative of at least three independent experiments. **(C)** Deoxynucleotides (NTPs) in whole cell lysates of WT and KO were quantified by DNA polymerase assay. The bar graph displays mean ± SEM from three independent experiments. **(D)** Intracellular citrate levels in WT and KO cells after 6 h treatment with PBS (UT) or hydroxycitrate (HC, 30 mM) quantified by LC-HRMS. The graph displays relative peak areas (a.u.) as mean ± SEM from three experiments. **(E)** Oxygen consumption rate (OCR) by WT or KO cells 2 h after HC (15 mM) injection by Seahorse XF instrument. Percent of OCR at the time of HC injection is presented as mean ± SEM from five independent experiments. **(F)** MICA surface expression on WT and KO cells analyzed by flow cytometry after 42 h treatment with indicated concentrations of HC. The bar graph displays isotype corrected MICA expression (ΔMFI) as mean ± SEM from three independent experiments. **(G)** MICA surface expression on WT and KO cells analyzed by flow cytometry after 18 h treatment with HC (15 mM) with or without indicated concentrations of oxaloacetate (OAA). ΔMFI is shown as percent of corresponding treatment without HC, and graph displays mean ± SEM from three independent experiments. **(H)** MICA surface expression on WT and KO cells 3 days post transfection with scrambled siRNA (siCtr), siIDH1 or siIDH2. The bar graph displays ΔMFI as mean ± SEM from three independent experiments. **(I)** MICA surface expression on WT and KO cells after 18 h treatment with indicated concentrations of citrate. The bar graph displays ΔMFI as mean ± SEM from three independent experiments. Statistical analysis was performed by unpaired *t*-test in **(C)**, two-way ANOVA with Bonferroni’s multiple comparison test in **(D–F,I,H)**, and one-way ANOVA with Dunnett’s multiple comparison test in **(G)**. *p < 0.05, **p < 0.01, and ***p < 0.001.

In a metabolite screen, we found that most detected and confirmed intracellular metabolites were more abundant in MGAT5 KO cells compared with WT cells (Figure 5A). When measuring forward scatter by flow cytometry, we furthermore found that MGAT5 KO cells were larger in size (Figure 5B). Additionally, MGAT5 KO cells had higher dNTP levels, specifically dTTP and dATP (Figure 5C). Combined with the lipid accumulation (Figure 4E), this indicates that MGAT5 KO cells accumulate metabolites compared with WT cells.

In contrast, citrate was the only metabolite that was lower in MGAT5 KO cells (Figure 5A). Cancer cells support increased growth and proliferation by exporting mitochondrial citrate to the cytoplasm. When citrate exits the mitochondria through the citrate/malate-exchanger, it can be converted to acetyl-CoA and OAA by the cytosolic ACLY. The citrate analog hydroxycitrate (HC) is a non-functional competitive inhibitor of ACLY. We found no difference in ACLY expression or the activating ACLY^*S*455^ phosphorylation (65) between WT and MGAT5 KO cells (Supplementary Figure S5A). However, treatment with HC resulted in citrate accumulation that was more pronounced in MGAT5 KO cells compared with WT cells (Figure 5D), suggesting that the lower levels of citrate in MGAT5 KO cells is caused by a high citrate turnover (Figures 5A,D). To further validate that MGAT5 KO cells had increased turnover of citrate, we blocked ACLY to inhibit citrate export from mitochondria, thereby forcing citrate to be oxidized in the TCA cycle. Indeed, only MGAT5 KO cells increased OCR significantly after HC treatment (Figure 5E). Moreover, HC reduced surface MICA expression in MGAT5 KO cells (Figure 5F), and this reduction was validated by another ACLY inhibitor BMS303141 (Supplementary Figure S5B). HC decreased MICA mRNA levels as well, suggesting a transcriptional regulation (Supplementary Figure S5C). OAA can readily be converted to malate and re-enter the mitochondria to keep the citrate/malate-exchanger running, and addition of OAA rescued the expression of MICA after ACLY inhibition (Figure 5G), suggesting that MICA expression depends on preserved flux of citrate from the mitochondria. Supporting this, we found that knockdown of isocitrate dehydrogenase 2 (IDH2) that converts isocitrate to α-ketoglutarate in the mitochondria, potentiated MICA expression in MGAT5 KO cells (Figure 5H and Supplementary Figure S5D). This indicates that increasing citrate efflux by blocking the TCA cycle downstream of citrate augments MICA expression. Furthermore, knockdown of the cytosolic IDH1 likewise upregulated MICA in MGAT5 KO cells (Figure 5H and Supplementary Figure S5D). Finally, MICA expression was amplified with the addition of citrate in MGAT5 KO cells but not WT cells (Figure 5I and Supplementary Figure S5E), indicating that the metabolic reprogramming in MGAT5 KO cells has provided a capacity to translate cytosolic citrate to MICA expression. Combined, this suggests that MICA expression in MGAT5 KO cells is maintained by mitochondrial efflux of citrate.

### Increased Chromatin Accessibility at the MICA Transcription Start Site in MGAT5 KO Cells

**FIGURE 6:**
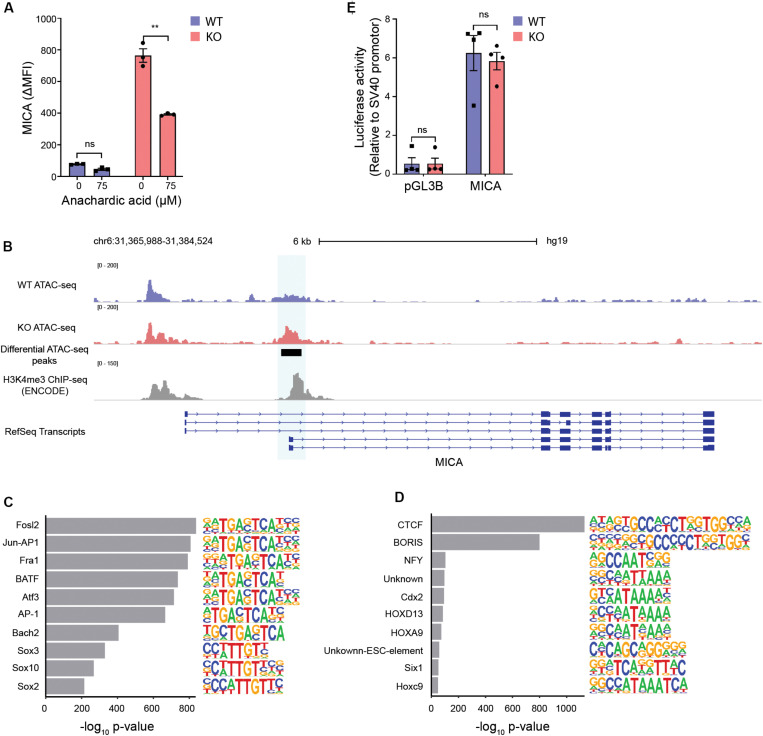
Increased chromatin accessibility at the MICA transcription start site in MGAT5 KO cells. **(A)** MICA surface expression analyzed by flow cytometry in HEK293 wildtype (WT) and HEK293 MGAT5 knockout (KO) cells after 24 h treatment with the HAT inhibitor anachardic acid (75 μM). The bar graph displays isotype corrected MICA expression (ΔMFI) as mean ± SEM from three independent experiments. **(B–D)** Chromatin accessibility in WT and KO cells analyzed by ATAC-seq. **(B)** Chromatin accessibility of WT and KO at chromosome 6 spanning the MICA gene. Normalized ATAC-seq data are aligned with MICA transcripts from RefSeq and ENCODE H3K4me3 ChIP-seq data from HEK293 cells. Genomic regions with significant differences in chromatin accessibility are highlighted in blue. The ATAC-seq track is representative of three independent experiments. **(C)** Top 10 enriched motifs in peaks upregulated in MGAT5 KO cells. **(D)** Top 10 enriched motifs in peaks downregulated in MGAT5 KO cells. **(E)** MICA promoter activity in WT and KO cells analyzed as luciferase activity 24 h after transfection with promoter-less firefly luciferase construct (pGL3B) or firefly luciferase-coupled MICA promoter (MICA). Firefly luciferase activity is normalized to an SV40 promoter-coupled renilla luciferase co-transfected into the cells. The bar graph displays mean ± SEM from four independent experiments. Statistics in **(A,E)** are analyzed using two-way ANOVA with Bonferroni’s multiple comparison test. **p < 0.01.

Conversion of citrate by ACLY regulates proliferation and gene expression by providing acetyl-CoA for fatty acid synthesis and histone acetylation (14). Inhibiting the synthesis of fatty acids did not impair MICA expression in MGAT5 KO cells (Supplementary Figure S6A). Similar to citrate, acetate can increase the acetyl-CoA pool and has previously been shown to induce MICA expression in cancer cells (15). In line with this, we found that acetate potentiated MICA expression in MGAT5 KO cells (Supplementary Figure S6B), indicating that MICA expression in MGAT5 KO cells could be responsive to increased acetyl-CoA. To test if MICA expression depended on protein acetylation, we inhibited histone acetyl transferases (HATs) with anachardic acid, and this impaired MICA expression in MGAT5 KO cells significantly (Figure 6A). Since histone acetylation regulates chromatin accessibility that modulates gene transcription, we next investigated if this was altered in MGAT5 KO cells. Indeed, ATAC-seq analysis revealed that MGAT5 KO cells had a region −196 to + 264 bp from the MICA transcription start site where the chromatin was significantly more open than in WT cells (Figure 6B), indicating increased accessibility for transcriptional activity. Analysis of chromatin accessibility across the whole genome demonstrated that several of the top 10 motifs that were enriched in MGAT5 KO cells were binding sites for factors related to the activating protein-1 (AP-1) transcription factor complex (Figure 6C) which is known to bind to the MICA promoter (66). Reciprocally, downregulated peaks comprised consensus motifs for the transcriptional repressor CTCC-binding factor (CTCF) and the CTCF-like Brother of the Regulator of Imprinted Sites (BORIS) that are known regulators of chromatin structure (Figure 6D) (67, 68). These findings suggest that CTCF-mediated chromatin condensation in WT cells may be abrogated in MGAT5 KO cells, possibly due to metabolite-mediated epigenetic changes.

The region of the MICA promoter that displays increased chromatin accessibility in MGAT5 KO cells is known to be involved in the transcriptional regulation of MICA and consistent with this, it is rich in H3K4me3 marks (Figure 6B). This region comprises binding sites for heat shock factor, Nuclear factor kappa-light-chain-enhancer of activated B cells (NF-κB), Sp1, and AP-1 transcription factor complex (Supplementary Figure S6C) (30, 43, 66). To investigate if MICA transcription was upregulated due to increased activity of transcription factors binding to this region, we measured luciferase activity in a MICA promoter construct expressed in WT or MGAT5 KO cells. The MICA promoter was constitutively active in both WT and MGAT5 KO cells compared with the promoter-less construct pGL3B, but luciferase activity was comparable in WT and MGAT5 KO cells (Figure 6E), indicating that the raised MICA transcript levels in MGAT5 KO cells are not driven by increased transcription factor activity at this site. Further, MICA promoter constructs with mutations in either NF-κB binding site, heat shock element (HSE) or both (30), were equally expressed in WT and MGAT5 KO cells (Supplementary Figure S6D). Histone acetylation is associated with open transcriptionally active chromatin and the histone deacetylase inhibitor FR901228 further upregulated MICA expression in MGAT5 KO cells (Supplementary Figure S6E). Transient stimulation with the short-chain fatty acid propionate also upregulated MICA expression in both WT and MGAT5 KO cells, indicating that acute stress-induced MICA regulation may be distinct from regulation of constitutively expressed MICA (Supplementary Figure S6E). These findings suggest that constitutive MICA expression in MGAT5 KO cells is influenced by the increased openness of the chromatin structure around the MICA transcription start site.

### Hydroxycitrate Reduces MICA Expression in Activated T Cells and Multiple Cancer Cells

**FIGURE 7:**
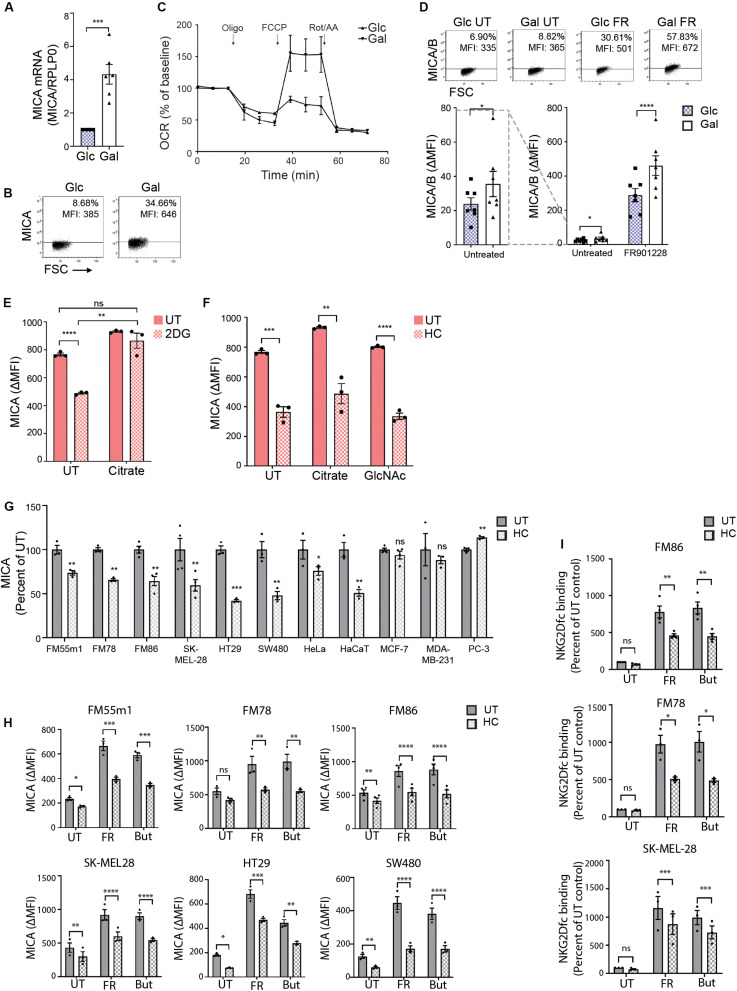
Hydroxycitrate reduces MICA expression in activated T cells and multiple cancer cells. **(A)** MICA mRNA analyzed by quantitative RT-PCR in total RNA purified from HEK293 cells after more than 30 passages in glucose (Glc) and galactose (Gal). MICA expression is normalized to housekeeping gene RPLP0 and displayed as mean ± SEM from six independent experiments. **(B)** MICA surface expression analyzed by flow cytometry of Glc and Gal cells at basal levels. Dot plots are representative of at least three independent experiments. Grid is set to ∼5% of corresponding isotype control stainings. **(C)** Mitochondrial stress test on HEK293 cultivated in Glc or Gal under same conditions as in Figure 4A. The graph is baselined to measuring point three and displays mean ± SEM from two independent experiments. **(D)** MICA/B surface expression of peripheral blood lymphocytes (PBLs) activated for 3 days in Glc or Gal growth medium prior to 18 h treatment with FR901228 (20 ng/mL). Grids in dot plots are set to ∼5% of corresponding isotype control staining and dot plots are representative of seven different donors. The bar graph displays mean ± SEM of isotype-corrected MICA/B MFI (ΔMFI) from seven donors. Left panel is zoomed in on the difference between untreated Glc and Gal PBLs. **(E,F)** HEK293 MGAT5 knockout (KO) cells were treated with (E) 2DG (20 mM) or **(F)** hydroxycitrate (HC) (15 mM) in addition to PBS (UT), citrate (10 mM), or GlcNAc (25 mM) for 22–24 h. Bar graphs display MICA surface expression as mean ± SEM of ΔMFI values from three independent experiments. Data of UT samples share values with UT samples in Figure 3H. **(G)** MICA surface expression in several cancer cell lines after 18 or 42 h treatment with HC (10 mM). ΔMFI values are normalized to UT control and shown as mean ± SEM from at least three independent experiments. **(H,I)** MICA surface expression **(H)** and NKG2D-fc binding **(I)** in cancer cell lines after 2.5 h treatment with HC (10 mM) prior to 18 h stimulation with FR901228 (FR, 20 ng/mL) or sodium butyrate (But, 5 mM). Bar graphs display MICA surface expression as mean ± SEM of ΔMFI values (H), or NKG2D-fc surface binding as ± SEM of ΔMFI normalized to untreated (UT) control **(I)**, from three independent experiments. Statistical analysis was performed by unpaired *t*-test with Welch’s correction in **(A,E,F)**, ratio paired *t*-test in **(D)**, one-sample *t*-test in **(G)**, and two-way ANOVA with Bonferroni’s multiple comparison test **(H,I)**. *p < 0.05, **p < 0.01, ***p < 0.001, and ****p < 0.0001.

An increasing variety of histone modifications has been reported to modify chromatin accessibility. A recent study demonstrated that lactate can be used for lactylation of histones and thus regulate gene transcription (69). Since MGAT5 KO cells had reduced secretion of lactate (Figures 3D,E), intracellular lactate could modify chromatin structure like acetyl-CoA. We tested if MICA expression in MGAT5 KO cells was predominantly dependent on changed glycolytic or mitochondrial function. To distinguish the effects, we forced WT cells to lower glycolysis and increase mitochondrial respiration by cultivation in galactose-medium as previously described (70–72) (Supplementary Figure S7A). Galactose is processed at a slower rate than glucose, and cells thereby favor mitochondrial respiration for energy production. Interestingly, WT cells in galactose increased MICA mRNA levels after long-term cultivation (over 30 passages) (Figure 7A). MICA surface expression; however, was only marginally upregulated (Figure 7B). This indicates that increased mitochondrial function can facilitate MICA transcription, but mitochondrial activity is not sufficient for upregulating MICA surface expression to the levels seen in MGAT5 KO cells, which also have functional glycolysis. Similar to MGAT5 KO cells, we found that WT cells cultivated in galactose medium had increased SRC (Figure 7C). Moreover, their MICA expression was impaired by treatment with HC (Supplementary Figure S7B) and amplified by treatment with citrate (Supplementary Figure S7C), supporting that cells cultivated in galactose have gained an enhanced capacity to express MICA, resembling MGAT5 KO cells.

Activated T cells are some of the few healthy cells that are capable of expressing MICA (73, 74). Furthermore, some T cell subsets shift to a cancer-like metabolism upon activation (75). We therefore investigated if expression of MICA/B by activated T cells was potentiated by enforcing mitochondrial activity with galactose-cultivation. We activated PBLs with CD3/CD28 and IL-2 for 3 days in glucose or galactose medium prior to stimulation with the HDACi FR901228. We observed an increase in basal MICA/B expression in galactose-cultivated PBLs as well as a prominent amplification of HDACi-induced MICA/B expression compared with glucose-cultivated PBLs (Figure 7D). This expression was blocked by HC (Supplementary Figure S7D). Combined, this indicates that the metabolic properties of galactose-cultivation supports constitutive MICA transcription, much like MGAT5 KO cells, supporting that mitochondria can drive basal MICA expression even at low glycolytic conditions.

In line with this, we found that MICA inhibition after blocking glycolysis in MGAT5 KO cells could be completely annulled by simultaneous addition of citrate (Figure 7E). In comparison, MICA expression after HC, could not be recovered by citrate or GlcNAc supplementation (Figure 7F), highlighting that citrate turnover is critical for MICA expression and distal to glycolytic regulation of MICA in MGAT5 KO cells. Interestingly, we also discovered that the NKG2DLs ULBP2/5/6, which were also upregulated in MGAT5 KO cells (Figure 1A), were not significantly regulated by modulating mitochondrial functionality, citrate metabolism, or histone acetylation, using etomoxir, HC or anachardic acid, respectively (Supplementary Figures S5F–H). To elucidate if cancer cells with constitutive MICA expression shared the same dependency on citrate conversion to maintain MICA expression, we treated different cancer cell lines with HC. Indeed, MICA expression on melanoma cell lines FM55m1, FM86, FM78, and SK-MEL-28, colorectal cancer cell lines HT29 and SW480, as well as HeLa and HaCaT cells were sensitive to ACLY inhibition by HC (Figure 7G). However, breast cancer cell lines MCF-7 and MDA-MB231, and prostate cancer cell lines PC-3 were either unresponsive to HC or increased MICA expression (Figure 7G). MICA expression in the HC-unresponsive MDA-MB231 cells were also unaffected by cultivation in galactose (Supplementary Figure S7E), suggesting that some cancer cells hold metabolic properties that have uncoupled MICA expression from citrate-metabolism. Upregulation of MICA on melanoma and colorectal cancer cell lines by treatment with HDACi FR901228 or butyrate, was significantly inhibited by HC treatment (Figure 7H), resulting in reduced binding of NKG2Dfc soluble receptor, as shown in FM78, FM86, and SK-MEL28 (Figure 7I). HC did not impair ICAM-1 expression, except form a minor reduction in FM55m1 and HT-29 cells (Supplementary Figure S7F), suggesting that citrate flux is essential for MICA expression in certain cancer cells.

Altogether, our data demonstrate that constitutively increased MICA expression in MGAT5 KO cells relied on metabolic alterations, and suggest that MICA expression was upregulated through an enhanced chromatin accessibility. A similar phenotype was observed in cancer cells that share a sensitivity to the ACLY inhibitor HC, and we propose that citrate drives basal MICA expression by providing acetyl-CoA for histone acetylation that can modify chromatin structure around the MICA transcription start site. Metabolic reprogramming of cancer cells can thus be monitored by the immune system through increased NKG2DL expression.

## Discussion

Altered metabolism is a recognized hallmark of cancer cells that has promised therapeutic opportunities for more than a decade (76). We have shown that citrate exported from mitochondria drives expression of MICA, a stress-induced ligand for the immune activating NKG2D receptor in multiple cancer cell lines, and we put forward a model where constitutive MICA expression is facilitated by metabolic regulation of chromatin structure.

Through examination of glycosylation-dependent MICA, we found that KO of the *N*-acetylglucosaminyltransferase MGAT5 increased MICA expression and thereby increased activation of NKG2D *in vitro* and *in vivo*. However, this is caused by altered metabolism rather than changes in MICA *N-*glycosylation. MICA^∗^008 and MICA^∗^018 are distinctly regulated by N-glycosylation, but both were expressed on the surface of both WT and MGAT5 KO cells, indicating that neither depend on complex N-glycan formation. HEK293 cells are homozygous for the MICA^∗^008 allele (77), and we found that MICA^∗^008 was upregulated posttranslationally in HEK293 MGAT5 KO cells, suggesting that there could be a contribution of allele-specific regulation. However, the posttranslational upregulation was not sufficient to describe the vast upregulation of endogenous MICA expression in MGAT5 KO cells was predominantly regulated transcriptionally. MGAT5 expression is in many cases associated with tumor progression and metastatic potential (22, 24, 78, 79). Inhibition of cancer-associated MGAT5 is considered a potential therapeutic target, and has led to immune activation in a breast cancer mouse model (27). In line with this, our data suggest that MGAT5 KO increases NKG2DLs, which would support cancer elimination in response to MGAT5 inhibition. Furthermore, MGAT5 KO has been associated with inflammatory diseases such as colitis (80), which also correlates with increased expression of NKG2DLs and thereby increased immune activation. Nevertheless, we found that MICA regulation was a long-term response to MGAT5 KO, hence, other early events may interfere and influence immunity before MICA expression is established. Further studies are required to pinpoint the direct involvement of MGAT5 and NKG2DLs in cancer and autoimmune diseases. This model; however, gives a framework for understanding constitutive regulation of NKG2DLs, and MGAT5 KO cells have here proven useful for investigating relevant cell metabolic features driving constitutive MICA expression.

Others have shown that MGAT5 overexpression increases surface expression of growth factor receptors through glycosylation and thereby regulate growth (25, 26, 56, 81). Notably, our MGAT5 KO cells also show increased growth and glucose uptake, possibly due to an excessive compensating feedback mechanism when adapting to the lack of MGAT5, which is likely driven by changes in the transcriptome, since glucose transporters were upregulated transcriptionally. We found that long-term UDP-GlcNAc accumulation in MGAT5 KO cells was important for MICA expression, which could contribute to the new metabolic phenotype. MGAT5 KO cells have increased aerobic glycolysis and lipid accumulation, suggesting enhanced lipid biosynthesis, compared with WT cells. These are all traits of cancer metabolism and could infer that MGAT5 KO cells resemble cancer cells metabolically (82).

Anti-cancer immune cells rely on some of the same metabolic pathways as cancer cells for exercising effector functions, and therapeutic strategies that target cancer metabolism can thus impede anti-cancer immunity (83). It is therefore critical to delineate metabolic pathways that are essential for cancer cells selectively or even promote immune surveillance. We found that metabolic changes in MGAT5 KO cells support MICA expression, since inhibition of glycolysis or disruption of mitochondrial integrity reduced chronic MICA expression. We argue that mitochondrial function is central for regulating constitutive MICA expression in MGAT5 KO cells and CD3/CD28-activated PBLs, since lowering glycolysis with culture medium deplete of glucose and supplemented with galactose raised MICA expression. Although constitutive MICA expression in MGAT5 KO cells was dependent on functional mitochondria, basal OXPHOS was similar in WT and MGAT5 KO cells, indicating that basal OXPHOS is not driving MICA expression.

Mitochondria export citrate to the cytosol for biosynthetic purposes, and this is increased in cancer cells (10). Cytosolic citrate inhibits phosphofructokinase of the glycolysis; therefore, maintaining a low level of cytosolic citrate is crucial for sustaining cancer-related aerobic glycolysis (13). We found that citrate flux was high in MGAT5 KO cells, maintaining low citrate levels that can support the increased aerobic glycolysis. The citrate efflux from mitochondria was critical for MICA expression in MGAT5 KO cells, and citrate supplementation could potentiate MICA expression. A cancer-associated mutation of IDH1 that generates the oncometabolite 2-hydroxyglutarate from cytoplasmic α-ketoglutarate (expected to diminish cytoplasmic citrate) has previously been shown to inhibit expression of the NKG2DLs ULBP1 and ULBP3 (84). This corresponds with our findings that cytoplasmic citrate is a driver of NKG2DL expression, and in further support of this we found that knockdown of IDH1 or IDH2 potentiated MICA expression in MGAT5 KO cells. Intracellular citrate is associated with cancer aggressiveness in prostate cancer cells and is a suggested biomarker for response to therapy (13, 85). Citrate administration sensitizes cancer cells to chemo therapy *in vitro* and therefore has an anti-cancer therapeutic potential (13, 86). Our data indicate that citrate may also contribute to tumor immune surveillance by upregulating NKG2DLs in metabolically reprogrammed cells, thus promoting an anti-tumor response. However, we found that MICA expression depended on citrate conversion to acetyl-CoA and OAA by ACLY. Furthermore, we found that acetate-induced MICA was impaired by ACLY inhibition (Supplementary Figure S6B), in line with previous findings showing that acetate can enter that TCA cycle and expand the acetyl-CoA pool through ACLY cleavage of citrate (87). ACLY is often overexpressed in cancer, and inhibition of ACLY is known to impair tumorigenesis, and impede cancer stemness and growth (11, 12, 88, 89). We did not detect increased expression levels of ACLY in MGAT5 KO cells compared with WT cells, suggesting that the citrate flux is driven by changes in metabolite levels possibly promoted by to the increased glucose uptake. We found that basal MICA expression in MGAT5 KO cells as well as several cancer cells, is downregulated in response to ACLY inhibition with HC. HC likewise impaired HDACi and SCFA-induced MICA in cancer cells. The consequences of constitutive MICA expression are poorly understood since it may have dual roles on anti-tumor immunity (90). Treating cancer with HC and thus reducing MICA expression can result in tumor immune evasion. Conversely, some cancers exhaust anti-cancer immunity by persistent high NKG2DL expression (4–7, 91), here HC treatment may be a selective treatment option. This hypothesis, however, needs further experimental verification, since the current study relies on *in vitro* findings. HC inhibition of MICA expression was selective to some tumors. Interestingly, prostate cancer cell line PC-3 was the only cell type that upregulated MICA in response to HC. Prostate cells are known to have atypical citrate metabolism because they produce and secrete large amount of citrate as a component of semen (92). Hence, to implement this in future treatment approaches it is important to define the metabolic prerequisites that couples citrate to MICA expression.

Mechanistically, ACLY regulates histone acetylation by controlling availability of acetyl-CoA that modulates chromatin accessibility and gene transcription. We show that MGAT5 KO cells have more open chromatin at the MICA transcription start site and that MICA expression was reduced by HAT inhibition, indicating that increased MICA mRNA is promoted by increased MICA transcription. Plasmid DNA does not form proper chromatin (93) And transcription of luciferase reporter plasmids was not upregulated in MGAT5 KO cells, indicating that MGAT5 KO-induced elevated MICA expression is maintained by increased chromatin accessibility rather than increased activation of transcription factors able to bind to this site. Constitutive MICA expression in MGAT5 KO cells may thus be driven by omnipresent transcription factors activating MICA due to the higher chromatin accessibility. It is well established that acetyl-CoA availability regulates global acetylation levels and gene expression (94). However, a mechanism controlling gene specificity has been unclear. After the discovery that acetyl-CoA-producing enzymes, including ACLY, can locate to the nucleus (95, 96), it has been proposed that localized production of acetyl-CoA facilitates site-specific acetylation (97). This was not explored in this study, but is a relevant course for future investigations.

Differences between expression of individual NKG2DLs in different cell types complicates the studies of NKG2DLs. It remains to be understood what determines if a cell can express NKG2DLs. Here, we provide evidence for a possible instructive role of the cell metabolism in regulating MICA, proposing that the permanent metabolic shift in cancer cells and activated T cells primes cells for MICA expression. Our data indicate that citrate holds a major regulatory role in basal MICA expression, likely by regulating chromatin accessibility through acetylation (Graphical abstract). It is noteworthy that the NKG2DLs ULBP2/5/6 likewise were highly upregulated in our cell model, but regulated in a distinct manner yet to be delineated. We recently published that the TCA intermediate fumarate upregulates ULBP2/5/6 in ROS-dependent manner (98), which together emphasize that the cell metabolism can convey a stress signal through NKG2DLs. Defining the metabolic control of NKG2DLs can help us design strategies targeting cancer metabolism without impairing immune surveillance. Furthermore, constitutive MICA expression is associated with increased inflammation and autoimmune diseases (99). This study investigates metabolic control of the immune stimulatory ligand MICA with a focus on metabolism as a hallmark of cancer. It is therefore tempting to speculate that metabolic control of constitutive MICA expression also is important in cases of metabolism-associated chronic inflammation.

## Data Availability Statement

The raw data supporting the conclusions of this article will be made available by the authors, without undue reservation.

## Ethics Statement

The animal study was reviewed and approved by the Animal Experimentation Inspectorate, Ministry of Environment and Food, Denmark.

## Author Contributions

SM, MMe, MMa, LA, and SS: conceptualization. SM, MMe, MMa, AB, SJ, MH, BA, CD, ES, ED, TH, DL, and LA: methodology. SM, BA, CD, ES, ED, and TH: formal analysis. SM, MMe, MMa, AB, SJ, MH, RH, BA, CD, ES, ED, and TH: investigation. CW, CO’C, HW, and SS: resources. SM and SS: writing – original draft. SM, MMe, RH, MMa, AB, SJ, MH, BA, CD, LR, ES, ZG-H, ED, CW, TH, DL, CO’C, HW, LA, and SS: writing – review and editing. SM and TH: visualization. SS: supervision. CD, CW, CO’C, HW, and SS: funding acquisition. All authors contributed to the article and approved the submitted version.

## Conflict of Interest

The authors declare that the research was conducted in the absence of any commercial or financial relationships that could be construed as a potential conflict of interest.

## Footnotes

1 https://www.encodeproject.org/
