# Supplementary material for: Cytoplasmic Citrate Flux Modulates the Immune Stimulatory NKG2D Ligand MICA in Cancer Cells
Source: Front Immunol. 2020 Aug 11;11:1968. doi: 10.3389/fimmu.2020.01968 (PMC7431954; doi:10.3389/fimmu.2020.01968)
Supplement: Supplementary file 1 [file Data_Sheet_1.docx]

***Supplementary Material***

# Supplementary Figures


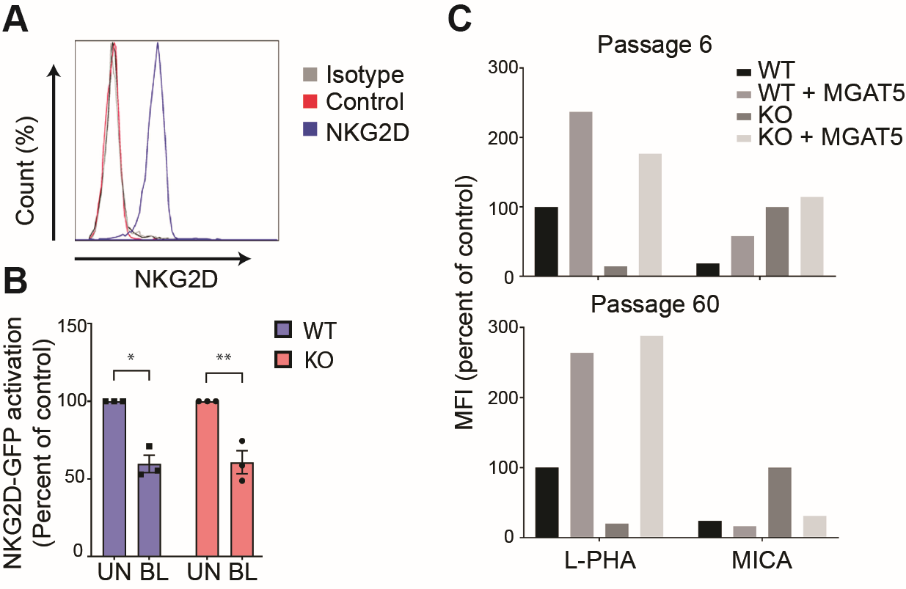


**Supplementary Figure 1**

**(A)** NKG2D surface expression on NKG2D-GFP reporter cell lines without NKG2D (Control) or with hNKG2D (NKG2D) analyzed by flow cytometry and shown as a histogram representative of at least three independent experiments. **(B)** NKG2D ligands on HEK293 wildtype (WT) and HEK293 MGAT5 knockout (KO) cells were blocked with soluble NKG2D-Fc (BL) or with control IgG1-Fc (UN) prior to co-cultivation with Control or NKG2D reporter cells in an effector:target ratio of 3:1 for 14-16 hours. GFP expression in NKG2D reporter cells was analyzed and mean ± SEM mean fluorescence intensity (MFI) values from three independent experiments are shown as peprcent of unblocked activation (UN). **(C)** L-PHA binding (MGAT5 modifications) and MICA surface expression after stable expression of MGAT5 into WT or KO cells. Analysis 6 and 60 passages after MGAT5 introduction are shown in upper and lower panel, respectively. MFI values for L-PHA binding is normalized to WT. MICA surface expression is normalized to KO basal expression. Statistical test performed in (B) was two-way ANOVA with Bonferroni's multiple comparison test. *p < 0.05, **p < 0.01 and ***p < 0.001


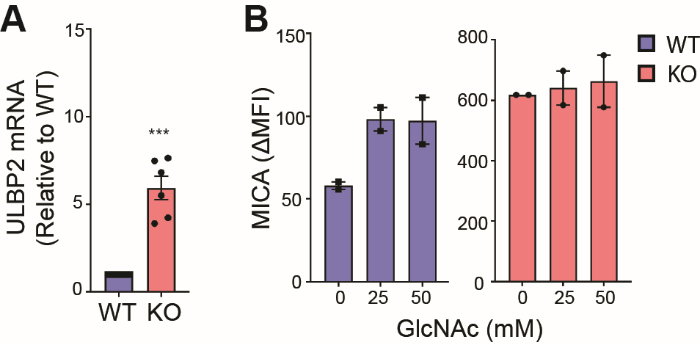


**Supplementary Figure 2**

**(A)** ULBP2 mRNAs were analyzed by quantitative RT-PCR in total RNA purified from HEK293 wildtype (WT) and HEK293 MGAT5 knockout (KO) cells. MICA expression was normalized to housekeeping gene RPLP0 and shown as ratio relative to WT cells mean ± SEM from six independent experiments. **(B)** MICA surface expression analyzed on WT and KO cells after 8 days cultivation with indicated concentrations of GlcNAc analyzed by flow cytometry. The bargraph shows mean ± SEM of isotype control-corrected MFI (ΔMFI) from two independent experiments. Statistical test performed in (A) was one-sample t-test. *p < 0.05, **p < 0.01 and ***p < 0.001


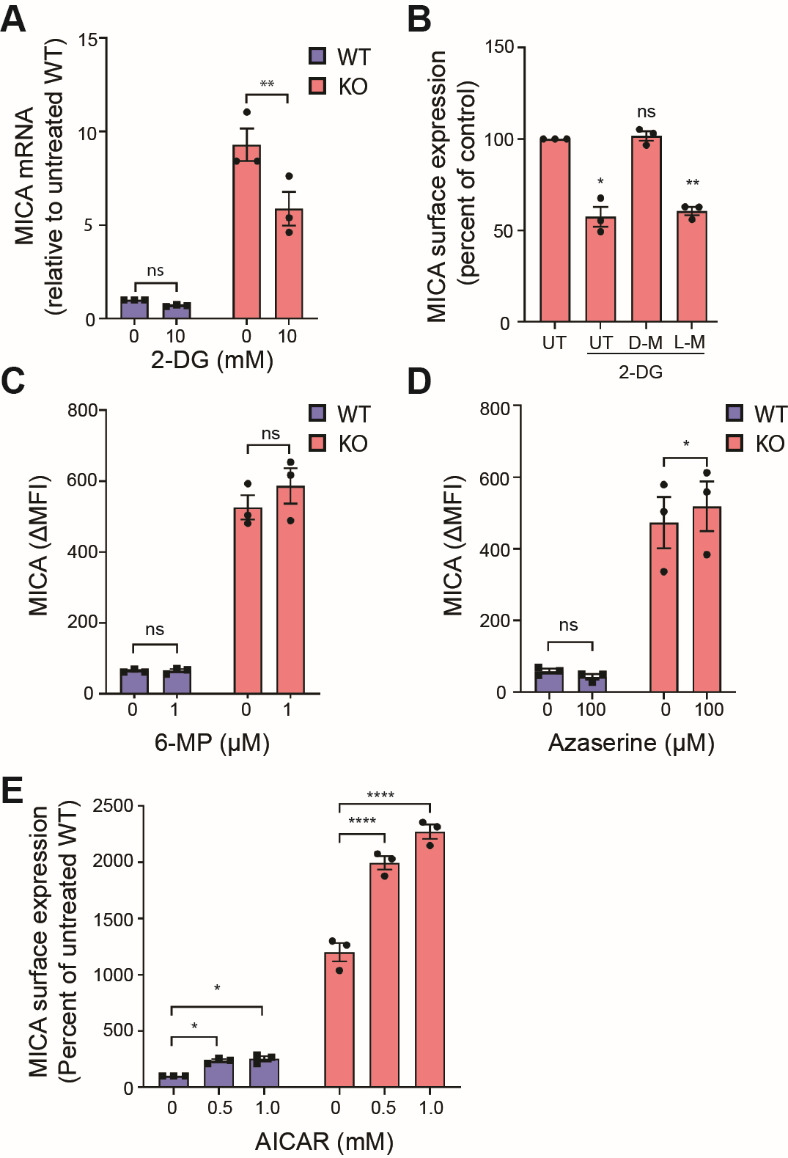


**Supplementary Figure 3**

**(A)** MICA mRNAs were analyzed by quantitative RT-PCR in total RNA purified from HEK293 wildtype (WT) and HEK293 MGAT5 knockout (KO) cells after 4 hours treatment with 2DG. MICA expression was normalized to housekeeping gene RPLP0 and shown as ratio relative to untreated WT cells. **(B)** MICA surface expression analyzed on KO cells after 18 hours with 20mM 2DG with or without 1mM D-Mannose (D-M) or L-Mannose (L-M), by flow cytometry. The bargraph shows mean ± SEM of MFI normalized to untreated sample. **(C-D)** MICA surface expression on WT and KO cells after 18 hours 6-mercaptopurine (6-MP) (C) or Azaserine (D) displayed as isotype-corrected surface expression (ΔMFI). (E) MICA surface expression on WT and KO cells after 20 hours AICA-R. Isotype-corrected surface expression (ΔMFI) is displayed as percent of untreated WT cells in each experiment. All graphs show mean ± SEM from three independent experiments. Statistical analysis was performed by two-way ANOVA with Bonferroni's multiple comparison test in (A), (C), (D) and (E), and one-sample t-test in (B). *p < 0.05, **p < 0.01 and ***p < 0.001


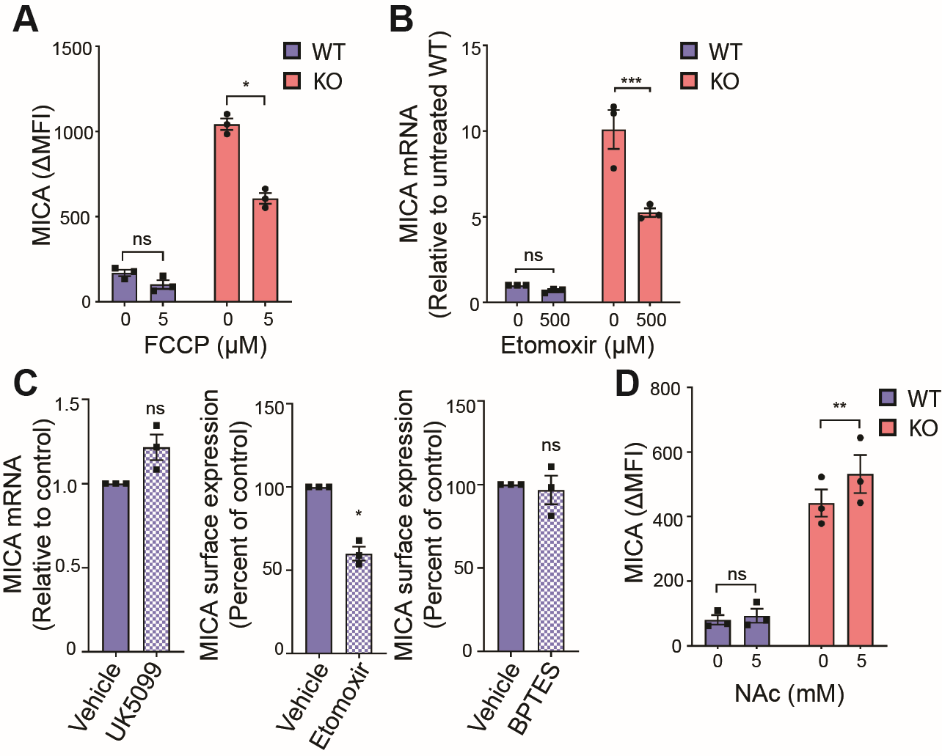


**Supplementary Figure 4**

**(A)** MICA surface expression on HEK293 wildtype (WT) and HEK293 MGAT5 knockout (KO) cells after 18 hours FCCP (5µM) analyzed by flow cytometry. The bargraph displays mean ± SEM isotype-corrected mean fluorescence intensity (ΔMFI) from three independent experiments. **(B)** MICA mRNAs were analyzed by quantitative RT-PCR in total RNA purified from WT and KO cells after 4 hours treatment with etomoxir (500µM). MICA expression was normalized to housekeeping gene RPLP0 and shown as ratio relative to untreated WT cells. The bargraph shows mean ± SEM from three independent experiments. **(C)** MICA expression after inhibition of TCA cycle substrates in WT cells: MICA mRNA analyzed by quantitative RT-PCR in total RNA purified from WT cells after 4 hours treatment with DMSO or the pyruvate import inhibitor UK5099 (200µM). MICA expression is normalized to housekeeping gene RPLP0 and displayed as ratio relative to DMSO control (left). MICA surface expresion analyzed by flow cytometry in WT cells after 18 hours treatment with fatty acid import inhibitor etomoxir (500µM) (middle) or glutaminolysis inhibitor BPTES (5µM) (right). ΔMFI values are displayed as percent of vehicle control. All three graphs display mean ± SEM from at three independent experiments. **(D)** MICA surface expression on WT and KO cells after 18 hours N-acetylcysteine (NAc), displayed as mean ± SEM ΔMFI from three independent experiments. Statistical analysis was performed by two-way ANOVA with Bonferroni's multiple comparison test in (A), (B) and (D), and one-sample t-test in (C). *p < 0.05, **p < 0.01 and ***p < 0.001


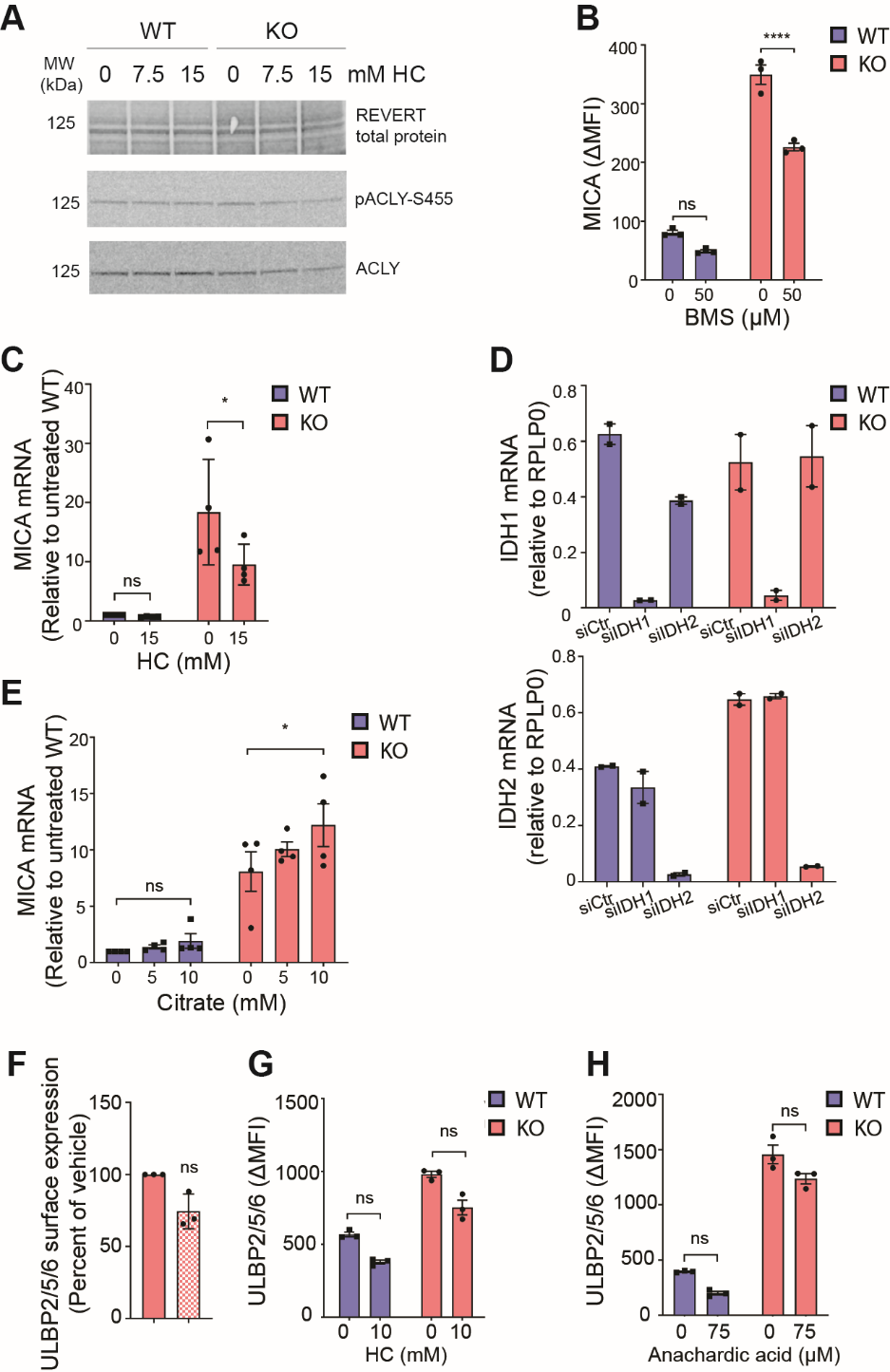


**Supplementary Figure 5**

**(A)** ATP citrate lyase (ACLY) and phospho-ACLY on Ser455 (pACLY-S455) with total protein stain control in whole cell lysates of HEK293 wildtype (WT) and HEK293 MGAT5 knockout (KO) after 2 hours treatment with hydroxycitrate (HC) at indicated concentrations. The blot is representatitve of three independent experiments. **(B)** MICA surface expression on WT and KO cells after treatment with ACLY inhibitor BMS303141 (BMS) for 42 hours analyzed by flow cytometry. The bargraph displays isotype-corrected mean fluorescence intensity (ΔMFI) as mean ± SEM from three independent experiments. **(C)** and **(E)** MICA mRNAs were analyzed by quantitative RT-PCR in total RNA purified from WT and KO cells after 4 hour treatment with HC (C), or citrate (E). MICA expression is normalized to housekeeping gene RPLP0 and displayed as ratio relative to untreated WT. The bargraphs show mean ± SEM from three independent experiments. **(D)** IDH1 and IDH2 mRNAs were analyzed by quantitative RT-PCR in total RNA purified from WT and KO cells 3 days post transfection with scrambled siRNA (siCtr), siIDH1 or siIDH2. IDH1/IDH2 expression was normalized to housekeeping gene RPLP0. The bargraphs show mean ± SEM from two independent experiments. (F-H) ULBP2/5/6 surface expression on WT and KO cells was analyzed by flow cytometry after 18 hour treatment with etomoxir (500µM) (F), 42 hours with HC (G), or 18 hours with anachardic acid (H). Statistical analysis in (C), (E), (G) and (H) was performed by two-way ANOVA with Bonferroni's multiple comparison test. One sample t-test was used in (F). *p < 0.05, **p < 0.01, ***p < 0.001, and ****p<0.0001


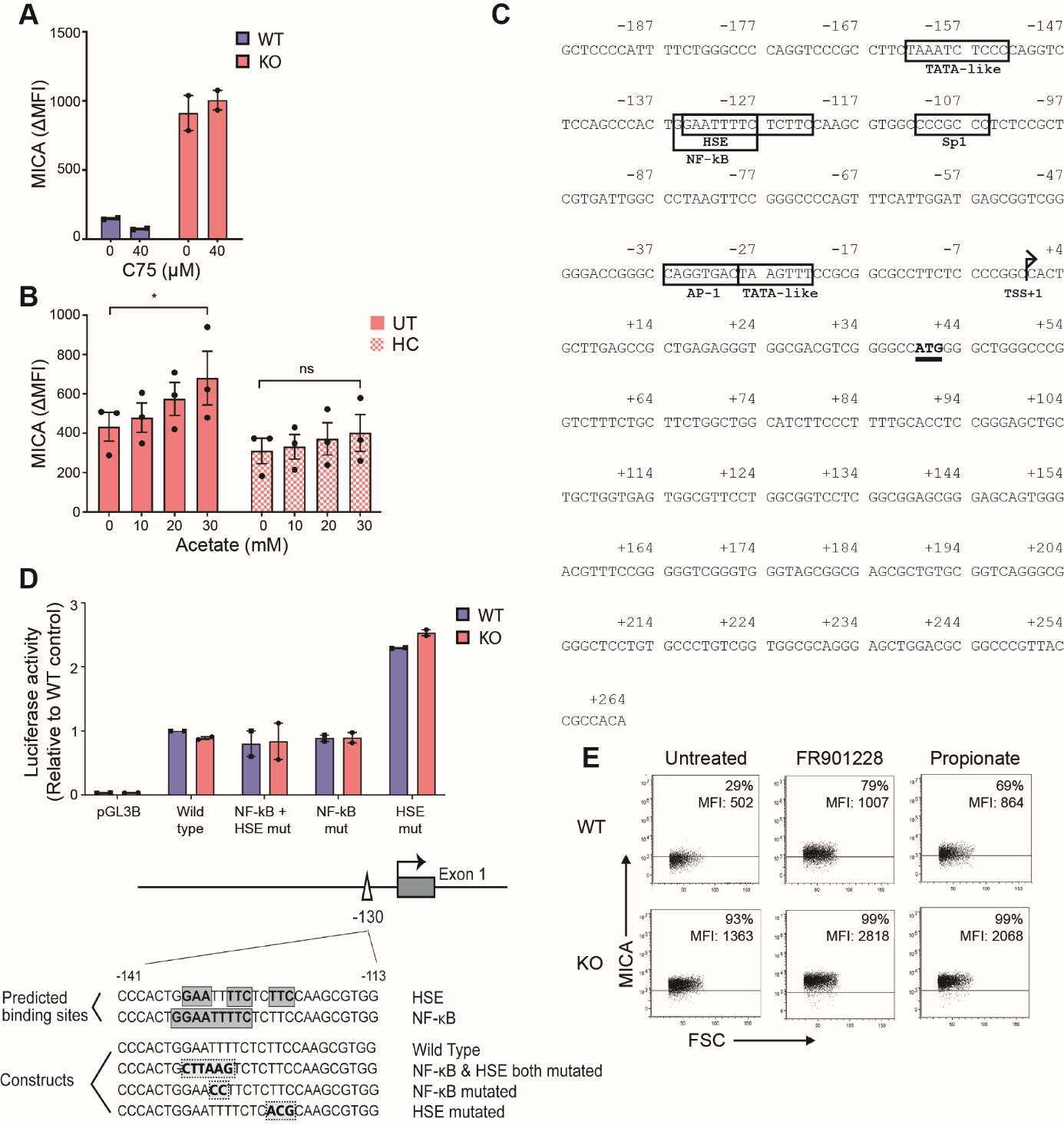


**Supplementary Figure 6**

**(A)** MICA surface expression on HEK293 wildtype (WT) and HEK293 MGAT5 knockout (KO) cells after 18 hours fatty acid synthase inhibitor C75 analyzed by flow cytometry. The bargraph displays isotype-corrected mean fluorescence intensity (ΔMFI) as mean ± SEM from two independent experiments. **(B)** MICA surface expression on KO cells after 18 hour treatment with acetate with or without 15mM hydroxycitrate (HC). The bargraph shows ΔMFI as mean ± SEM from three independent experiments.**(C)** Nucleotide sequence of the MICA gene *from -196 to +264*bp spanning MICA transcription start site (TSS) extracted from hg19 *Chr6:* 31,371,175-31,371,631. Known transcription factor binding sites are noted in boxes and the start codon is underlined in bold. **(D)** MICA promoter activity in WT and KO cells analyzed as luciferase activity 24 hours after transfection with promoter-less firefly luciferase construct (pGL3B) or firefly luciferase-coupled MICA promoter with mutations as shown in the schematic overview (Lin et al., 2012). Firefly luciferase activity is normalized to an SV40 promoter-coupled renilla luciferase co-transfected into the cells. The bargraph displays mean ± SEM from two independent experiments. **(E)** MICA surface expression on WT and KO cells after 18 hour treatment with FR901228 (20ng/mL) or propionate (10mM). Grids are set to 5% of the corresponding isotype control. Dot plots are representative of at least three independent experiments. Statistics in (B) were calculated by two-way ANOVA with Bonferroni's multiple comparison test.*p < 0.05, **p < 0.01 and ***p < 0.001


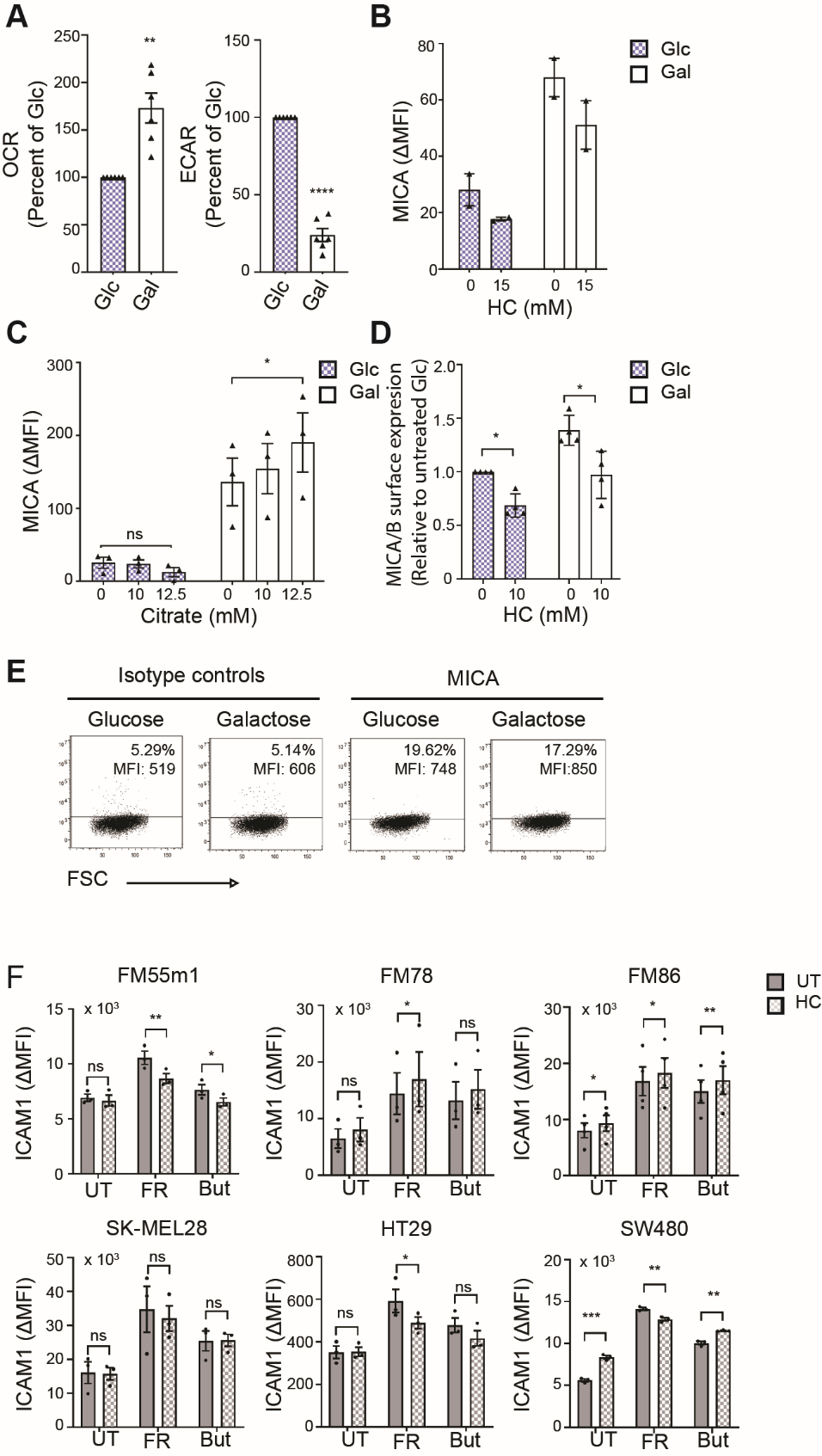


**Supplementary Figure 7**

**(A)** Oxygen consumption rate (OCR) and extracellular acidification rate (ECAR) in HEK293 cells cultivated in glucose (Glc) or galactose (Gal) analyzed by the Seahorse XF instrument. OCR and ECAR at baseline measurement point 3 are presented as percent of Glc. Bargraphs show mean ± SEM from six independent experiments. **(B-C)** MICA surface expression on HEK293 Glc and Gal cells after 18 hour treatment with hydroxycitrate (HC) **(B)** or citrate **(C)** analyzed by flow cytometry. The bargraphs display isotype-corrected mean fluorescence intensity (ΔMFI) as mean ± SEM from two or three independent experiments. **(D)** MICA/B surface expression on peripheral blood lymphocytes activated with CD3/CD28 for 3 days in Glc or Gal prior to 18 hour treatment with HC. The bargraph displays mean ± SEM of ΔMFI values normalized to untreated Glc from four donors. **(E)** MICA surface expresion on MDA-MB231 cells cultivated for 15 passages in Glc or Gal growth medium. Grids are set to ~5% of the corresponding isotype controls. **(F)** ICAM-1 surface expression in cancer cell lines after 2.5h treatment with HC (10mM) prior to 18h stimulation with FR901228 (FR, 20ng/mL) or sodium butyrate (But, 5mM). Bargraphs display ICAM-1 surface expression as mean ± SEM of ΔMFI values from three independent experiments. Statistical analysis was performed by one-sample t-test in (A), and two-way ANOVA with Bonferroni's multiple comparison test in (C), (D) and (F).*p < 0.05, **p < 0.01 and ***p < 0.001

**References**

LIN, D., LAVENDER, H., SOILLEUX, E. J. & O'CALLAGHAN, C. A. 2012. NF-kappaB regulates MICA gene transcription in endothelial cell through a genetically inhibitable control site. *J Biol Chem,* 287**,** 4299-310.
